# The low-recombining pericentromeric region of barley restricts gene diversity and evolution but not gene expression

**DOI:** 10.1111/tpj.12600

**Published:** 2014-08-05

**Authors:** Katie Baker, Micha Bayer, Nicola Cook, Steven Dreißig, Taniya Dhillon, Joanne Russell, Pete E Hedley, Jenny Morris, Luke Ramsay, Isabelle Colas, Robbie Waugh, Brian Steffenson, Iain Milne, Gordon Stephen, David Marshall, Andrew J Flavell

**Affiliations:** 1 University of Dundee at JHI, InvergowrieDundee, DD2 5DA, UK; 2 James Hutton Institute, InvergowrieDundee, DD2 5DA, UK; 3 Institute of Agricultural and Nutritional Sciences, Martin-Luther-University Halle-Wittenberg06120, Halle, Germany; 4 Department of Plant Pathology, University of MinnesotaSt. Paul, MN, 55108, USA

**Keywords:** barley, *Hordeum vulgare*, heterochromatin, genome evolution, pericentromeric

## Abstract

The low-recombining pericentromeric region of the barley genome contains roughly a quarter of the genes of the species, embedded in low-recombining DNA that is rich in repeats and repressive chromatin signatures. We have investigated the effects of pericentromeric region residency upon the expression, diversity and evolution of these genes. We observe no significant difference in average transcript level or developmental RNA specificity between the barley pericentromeric region and the rest of the genome. In contrast, all of the evolutionary parameters studied here show evidence of compromised gene evolution in this region. First, genes within the pericentromeric region of wild barley show reduced diversity and significantly weakened purifying selection compared with the rest of the genome. Second, gene duplicates (ohnolog pairs) derived from the cereal whole-genome duplication event ca. 60MYa have been completely eliminated from the barley pericentromeric region. Third, local gene duplication in the pericentromeric region is reduced by 29% relative to the rest of the genome. Thus, the pericentromeric region of barley is a permissive environment for gene expression but has restricted gene evolution in a sizeable fraction of barley's genes.

## Introduction

Barley was domesticated ca. 8000 years ago from its wild progenitor *Hordeum vulgare* ssp. *spontaneum* (hereafter termed *H. spontaneum*). It is the fourth most important cereal worldwide, after maize, rice and wheat. Barley is an inbreeding diploid species and has become a model for genomic research in other Triticeae crops, including wheat and rye. The sequence of the barley gene space, together with a framework for the genome sequence (International Barley Genome Sequencing Consortium (IBGSC), 2012); comprises 26 159 high-confidence (HC) genes anchored to a 3479-point genetic map (Comadran *et al*., 2012). The comparative genomics of barley versus small genome grass ‘model’ species has been analysed in depth and the major segmental rearrangements that distinguish the different genomes are known (Salse *et al*., 2008; Thiel *et al*., 2009; IBGSC, 2012).

The ancestor of the cereal grasses underwent a whole-genome duplication (WGD) event around 50–70 MYa (Salse *et al*., 2008). Since then there have been multiple lineage-specific genomic rearrangements (Salse *et al*., 2008; Thiel *et al*., 2009) in the evolving cereal lineages. In addition, there has been extensive gene loss, which has been biased in favour of one or other of the progenitor diploid genomes (Schnable *et al*., 2012).

Many cereal genomes are inflated in size, mainly as a result of the proliferation of transposable element (TE) insertions, most of which are retrotransposons (Paterson *et al*., 2009; Schnable *et al*., 2009; IBGSC, 2012). These insertions are more common in the regions surrounding the centromeres, leading to inflation in the pericentromeric (PC) region, which comprises at least 48% of the barley physical genome, containing an estimated 14–22% of the total barley gene content (IBGSC, 2012). Thus, gene number is high for the PC region and gene density is low, with each gene typically surrounded by huge TE arrays. This situation is not confined to cereals; for example, roughly 57% of the soybean genome and ˜22% of its genes are PC (Schmutz *et al*., 2010).

Recombination is reduced in the vicinity of TEs (Fu *et al*., 2002) and in PC regions it is strongly suppressed (Schnable *et al*., 2009; Schmutz *et al*., 2010; IBGSC, 2012; Higgins *et al*., 2012). In barley, recombination commences at telomeres and progresses internally with crossover interference inhibiting this process in the interior (Higgins *et al*., 2012). Lack of recombination in the PC regions renders the genes within it relatively inaccessible to breeders, who need to re-assort alleles to achieve crop improvement. Restricted recombination also has potential impact upon gene evolution (Begun and Aquadro, 1992; Hudson, 1994; Nordborg *et al*., 2005; Wright *et al*., 2006; Charlesworth *et al*., 2009). Multi-gene haplotypes in low-recombining (LR) regions are expected to evolve as concerted units with low diversity, which should be further reduced by the preferred selfing habit of most cereal crop species, including barley. Newly arising mutations in genes within LR regions, most of which would be either neutral or weakly deleterious, persist for many generations in close genetic linkage because recombination cannot separate them and selection cannot remove them. This phenomenon is known as Hill-Robertson interference.

The LR-PC region is predominantly heterochromatic, being highly compacted throughout the cell cycle. For sorghum, chromatin compaction and recombination rate are linked (Kim *et al*., 2005). In *Arabidopsis* DNA methylation and repressive histone covalent modifications such as H3K9Me2 (Lippman *et al*., 2004; Hall *et al*., 2012) correspond closely. For cereals there tends to be higher levels of repressive epigenetic marks in the PC regions but also clear evidence for the presence of such marks across the genome (Houben *et al*., 2003; Shi and Dawe, 2006; Carchilan *et al*., 2007; Higgins *et al*., 2012), consistent with the corresponding genomic distribution of retrotransposon insertions (Schnable *et al*., 2009; Paterson *et al*., 2009; IBGSC, 2012). Collectively, these data are consistent with the model that heterochromatin at the local level is defined by TE density (Lippman *et al*., 2004) and for cereals may potentially be found almost anywhere in the genome.

In animals, the juxtapositioning of heterochromatin near genes can lead to suppressed gene expression (Jost *et al*., 2012) but, in *A. thaliana* at least, genes surrounded by heterochromatin are insulated from heterochromatic silencing (Lippman *et al*., 2004). Total mRNA levels have been reported to be lower in the LR-PC region than for the predominantly high-recombining (HR) chromosome arms in soybean (Du *et al*., 2012) and maize (Gent *et al*., 2012). For rice, apparently contradictory results have been seen between chromosomes for averaged mRNA levels for the LR-PC versus HR regions (Yin *et al*., 2008; Wu *et al*., 2011). This issue is further complicated by the problem of gene annotation in the LR region, which contains decayed TE remnants that can be miss-annotated as ‘normal’ genes and thus inflate the apparent genic diversity of the PC region and distort other parameters such as gene expression data. In summary, the available evidence suggests that total mRNA levels are low across plant PC regions but this may be mainly due to low gene density, with averaged expression levels per gene not dramatically different from the rest of the genome.

LR-PC region residency also affects gain and loss of genes. Levels of gene tandem duplication in rice and *Arabidopsis* are correlated with recombination rate (Rizzon *et al*., 2006), as the former relies upon unequal exchange. Thus, multi-copy gene clusters would be expected to be smaller and/or less frequent in Triticeae LR DNA. Furthermore, genes that have become duplicated following segmental duplication or WGD events tend to be eliminated relatively rapidly in a process termed diploidization (Wolfe, 2001). Loss of WGD-derived gene paralogs (ohnologs [Wolfe, 2000]) has tended to occur asymmetrically among duplicated genome segments (Thomas *et al*., 2006; Woodhouse *et al*., 2010; Du *et al*., 2012), with HR–HR WGD gene duplicates having evolved more rapidly (Du *et al*., 2012). Retained gene pairs seem to diverge rapidly soon after duplication (Lynch and Conery, 2000) but more slowly than single genes overall (Yang *et al*., 2003; Yang and Gaut, 2011; Du *et al*., 2012).

In the current study, we have explored the diversity, evolution, expression and duplication of the genes in the LR-PC region of barley. We have compared these parameters to the rest of the genes of the species to discover how this genomic environment impacts upon them.

## Results

### Defining the low-recombining pericentromeric region of barley

**Figure 1:**
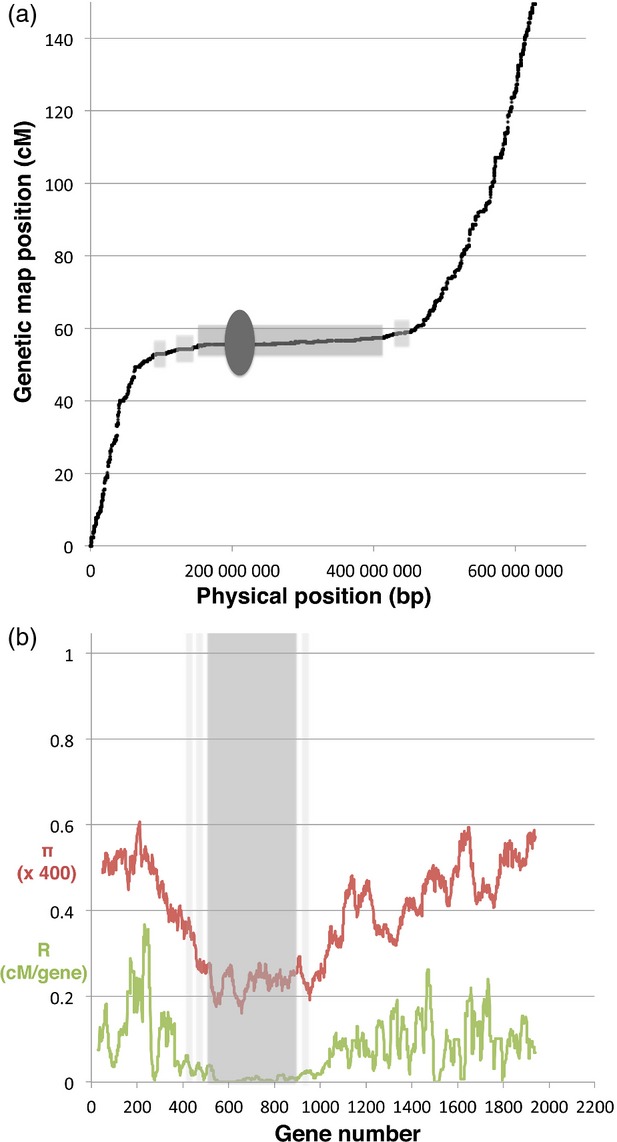
The LR-PC region of barley – definition, diversity and recombination. (a) Genetic versus physical map locations of genes on barley chromosome 2H. The centromere (dark grey oval) is surrounded by a continuous LR-PCH region (mid-grey bar), with flanking LR regions shown in light grey. (b) Diversity and recombination statistics for chromosome 2H. Rolling averages for gene nucleotide diversity (π, red) are plotted with recombination rate (cM/gene, green) against gene order. The LR-PCH regions (grey shading) correspond to the regions in (a).

The LR-PC region of barley can be visualised by plotting genetic map positions of genes or markers against their corresponding physical positions (Figures 1, S1 and Table S1). We define the LR-PC region as the continuous region surrounding the centromere for which recombination rate is 20-fold lower than the average for the barley genome (Choo, 1998). By this definition 6285 of 35 134 mapped barley genes (17.9%) are within the LR-PC region. If substantial LR regions flanking the PC region are included (Figure 1), a further 2400 genes (6.8%) are assigned to LR DNA, adding up to 24.7% of the total barley gene complement.

### Gene-based diversity in the LR-PC region of wild barley

To investigate gene sequence diversity across the genome of the wild species *H. spontaneum*, we selected 14 diverse wild barley samples (Figure S2; Experimental procedures). RNA-seq was performed on whole seedlings and sequence reads were mapped onto a consensus set of 22 651 full-length (FL) barley cDNAs (Matsumoto *et al*., 2011). Unsurprisingly, read-depth varied both within and between the FLcDNAs, since mRNAs are expressed at different levels with multiple splice variants and cDNA synthesis efficiency varies along its template. Fortunately, read depths for most positions of most genes were remarkably similar for different samples, giving good overlap for SNP discovery. Our mapping contained a total of 128 749 SNPs.

To estimate regional changes in gene diversity within the barley genome, nucleotide diversity (π) statistics for the mapped genes corresponding to the FLcDNAs were plotted against corresponding map positions (Figures 1b and S3). There is a marked drop in π in the interior of all seven chromosomes. When recombination rate is plotted on the same graphs it is evident that gene nucleotide diversity for *H. spontaneum* broadly follows recombination rate (R), with a pronounced drop in the vicinity of the LR-PC regions.

### mRNA expression levels in the LR-PC region

**Figure 2:**
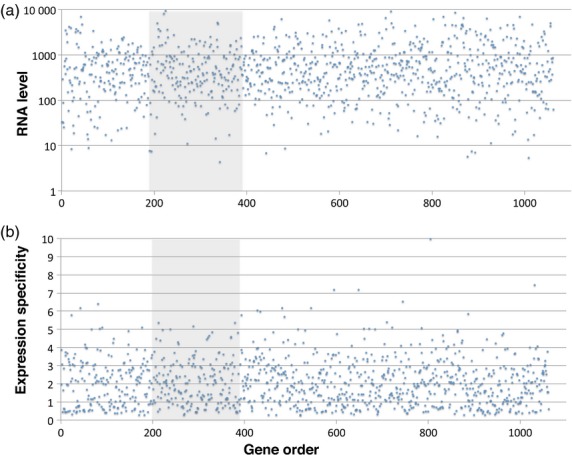
Gene expression level and developmental specificity are independent of LR-PC region residency in barley. Expression parameters for barley genes (Y axes) are plotted against their linear order (X axes) on barley chromosome 1H. The continuous LR-PCH is shaded grey. (a) Average RNA levels (arbitrary units), taken across 15 tissue types and developmental stages (Druka *et al*., 2006). (b) Developmental and/or tissue specificity quotients (= data from (a) divided by their corresponding standard deviations).

The LR-PC region has been considered a repressive environment and gene expression has reported to be reduced in the LR-PC region of maize and soybean (Du *et al*., 2012; Gent *et al*., 2012). We therefore explored mRNA expression across the barley genome. Steady-state mRNA levels in 15 tissue types, covering a variety of developmental stages of cv. Morex (Druka *et al*., 2006), were plotted against corresponding pseudo-physical map positions (IBGSC, 2012). Averaged RNA values across all tissue types are shown for chromosome 1H as an example in Figure 2(a) and detailed expression data are in Figure S4. No difference in RNA level was observed between the genes of the LR-PC region and those from the HR region, either by chromosome or by tissue type (anova, *P *> 0.05).

We also searched for possible differences in RNA expression specificity between the LR-PC and HR regions. Fluctuations between RNA levels for each gene among the 15 tissue and developmental stage types, relative to their corresponding average value (i.e. [average expression/standard deviation]; Figure 2b) were plotted. Again, no trend was visible, none was supported by anova (*P *> 0.05) and we conclude that the genes within the LR-PC regions show no significant evidence for differential expression specificity, relative to the rest of the genome.

### Gene evolution in the LR-PC region

**Figure 3:**
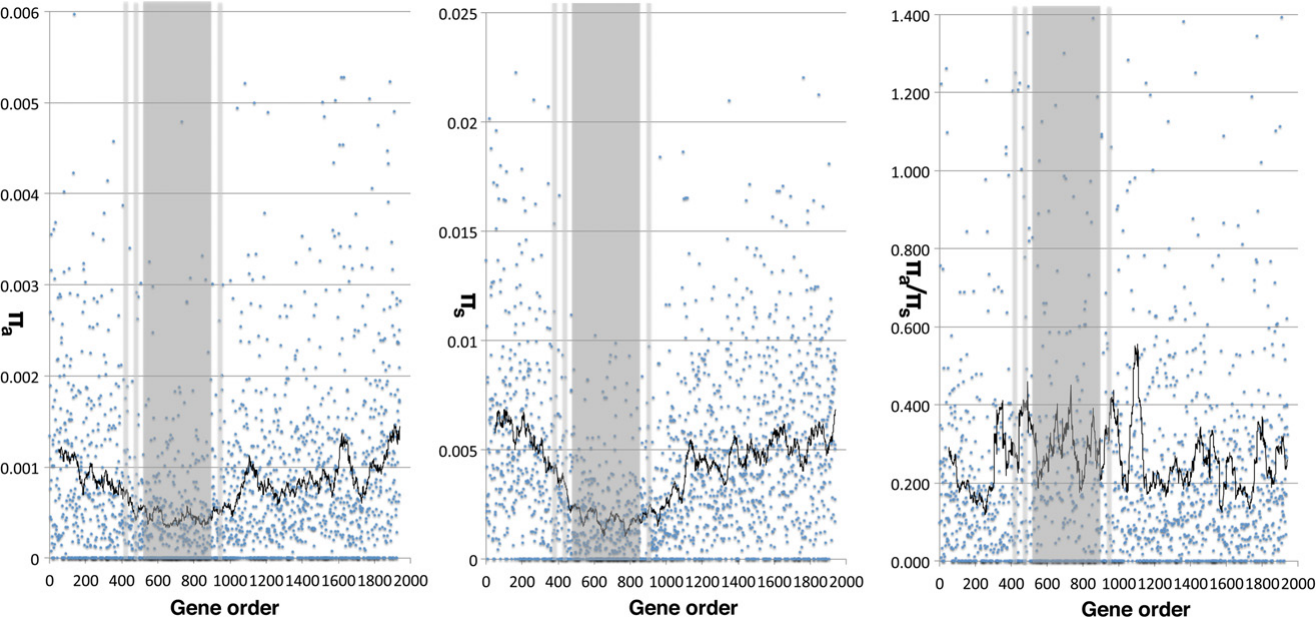
Gene selection is less effective in the LR-PC region than the HR genome compartment of *H. spontaneum*. π_a_, π_s_ and π_a_/π_s_ values per gene among 14 diverse *H. spontaneum* lines (Y axes) are plotted against their corresponding linear gene orders (X axes) on barley chromosome 2H. Black lines indicate rolling averages (50 genes) and the LR-PC regions are indicated by shading.

Restricted recombination within the LR-PC region is expected to impact upon its gene evolution (see Introduction), leading to increased non-synonymous substitution (π_a_), relative to the synonymous rate (π_s_) (Charlesworth *et al*., 2009). To test this prediction, gene-based π_a_, π_s_ and π_a_/π_s_ values were explored in wild barley (*H. spontaneum*). π statistics were determined for 5475 mapped genes in the 14 wild barley sample RNA-seq data set described above (Figures 3 and S5). Both π_a_ and π_s_ show marked diversity reduction in the LR-PC region and this effect is less pronounced for π_a_, consistent with the above predictions. π_a_/π_s_ plots show strong fluctuations, making it difficult to discern LR-PC effects for some chromosomes but the mean π_a_/π_s_ value for all LR-PC region genes is 0.235 (SD 0.010) and the same parameter for HR genes is 0.170 (SD 0.011; Table S2). This difference is significant (independent *t*-test; *t* = −11.507, df = 12, *P* < 0.001) and we conclude that the evolution of genes within the LR-PC region of *H. spontaneum* has been significantly impacted by Hill–Robertson interference.

### The LR-PC region and the cereal whole-genome duplication

To explore the effect of restricted recombination upon gene and genome evolution over the timescale of the evolution of cereal grasses, we searched for barley ohnolog gene pairs deriving from the cereal WGD that occurred *ca*. 60 MYa. Each gene in such pairs resides either in the LR-PC or the HR regions and the long-term effects of the two genome compartments upon gene evolution can be compared (see Discussion).

It is difficult to isolate WGD-derived barley ohnolog pairs because the Triticeae lineage has experienced high levels of translocation of genes and pseudogenes (Wicker *et al*., 2011) and local chromosome rearrangements, particularly segmental inversions (Luo *et al*., 2009) (Figure S6). We therefore adopted an iterative strategy designed to collect gene pairs showing sequence similarity within the broad range expected for WGD-derived duplication and genomic locations inherited from the WGD (i.e. no gene transposition). First, all barley genes with assigned genomic positions were compared against each other by blast search. Second, the output was filtered to remove gene pairs much too similar (i.e. recently duplicated) or diverged (>60 MYa separation) to be WGD-derived, Third, all genes in non-syntenic genomic positions relative to the rice, *Brachypodium* and sorghum genomes were removed, reasoning that a barley gene with no synteny support from these other cereal genomes is extremely unlikely to show synteny conservation from the WGD. Fourth, remaining barley genes were reordered according to local *Brachypodium* gene order, i.e. replacing barley genetic map order by Brachypodium physical order within collinear barley-Brachypodium synteny blocks (Figure S7). Finally, residual non-collinear barley genes with anomalous barley genetic map positions off the main orthology trend were removed (Figure S8).

This procedure yielded a final gene list of 12 348 mapped barley–*Brachypodium* syntenic gene pairs occupying substantially orthologous genomic locations between the two complete genomes. A chromosome versus chromosome X–Y plot of best BLAST hits with this cleaned-up set of barley genes revealed seven major WGD segments reported previously (Salse *et al*., 2008; Thiel *et al*., 2009; Figure S9) but the loose structure made it difficult to discriminate genuine ohnolog pairs from chance juxtaposition of transposed paralogs. We therefore used two approaches in parallel to eliminate the latter and our final selection represents a synthesis of both. First, the X–Y plots were manually edited to remove all pairs falling outside dense groupings, yielding 408 ohnolog pairs (Figure S10a). Second, the unedited paralog gene list was inputted into MCScanX (Wang *et al*., 2012), which finds ohnologous regions in genomes with ancient WGDs, yielding 366 pairs (Figure S10b). Merging these two lists yielded a consolidated candidate set of 498 ohnolog pairs (overlap = 276 gene pairs). Final manual trimming of this set removed 101 pairs with Ks scores >3, 107 pairs with genes involved in >1 pair, pairs in ohnologous regions comprising <9 genes and pairs in regions overlapping stronger regions of paralogy.

Our final list comprises 290 HC WGD ohnolog pairs (*= *580 genes) (Table S3, Figure S10c). 281 of these pairs are distributed among the seven previously defined WGD descendant chromosome segments (Salse *et al*., 2008; Thiel *et al*., 2009) and the remaining nine derive from the diploid cereal ancestral A3/A7 chromosome duplication (= barley 2H/5H).

### Properties of WGD-derived ohnolog pairs

If the LR-PC region has affected the properties of genes contained within it then there should be corresponding differences between the three possible classes of ohnolog pairs, namely LR–LR, LR–HR and HR–HR. The properties of the WGD ohnolog pairs retained by barley are summarized in Table 1.

**Table 1:** Distribution of barley ohnologs and ohnolog pairs by genome compartment and chromosome

Type	Genome compartment		Number									
			Obs^a^	Exp^a^	Chromosomal distribution							Total
					1H	2H	3H	4H	5H	6H	7H	
Ohnolog	HR		477	437	124	70	97	13	28	75	70	477
	LR		103	143	1	8	17	8	0	52	17	103
	HR–HR		187	164	107	38	97	12	3	75	42	374
Ohnolog pair	LR–HR	LR	103	107	1	8	17	8	0	52	17	103
		HR	103	107	17	32	0	1	25	0	28	103
	LR–LR		0	18	0	0	0	0	0	0	0	0

a Exp, Expected; Obs, Observed (see text).

Of the 580 ohnologs, 103 (18%) reside in the LR-PC region, together with flanking LR regions, compared to an overall gene content for this compartment of 25%. Thus, ohnologs have been preferentially lost from the LR-PC region (χ^2^ = 16.2, df = 1, *P* = 0.00006). When the distribution of the 290 ohnolog pairs among the three combinations of genome compartment (LR–LR, LR–HR, HR–HR) is examined, the main source of the loss becomes apparent. One hundred and eighty-seven ohnolog pairs are HR–HR (expected 164), 103 pairs are LR–HR (expected 107) and surprisingly, none are LR–LR (expected 18). This biased distribution is also highly significant (χ^2^ = 21.9, df = 2, *P* < 0.0001) and the main source of this is the LR–LR category. We conclude from these results that LR–LR WGD-gene pairs have been strongly selected against in the barley lineage and HR–HR and LR–HR classes show no significant evidence for this.

**Figure 4:**
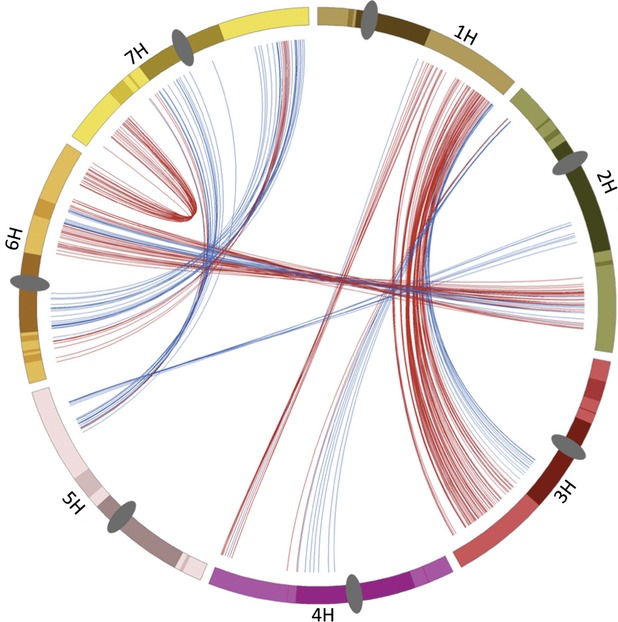
Barley ohnolog pairs and the LR-PC region. Barley chromosomes 1H–7H are indicated by different coloured bars, LR-PCH regions are indicated by dark shading, flanking LR by light shading and centromeres by dark grey ovals. All features are scaled by pseudo-physical map position (IBGSC, 2012). Ohnolog gene pairs are connected by colour-coded lines to indicate HR–HR pairs (red) and LR–HR pairs (blue). There are no LR–LR pairs.

The ohnologs are distributed unevenly across the genome (Table 1 and Figure 4). Chromosomes 4 and 5 carry only 10% of the total ohnologs within 27% of the total mapped gene content of barley, half of the entire LR-PC region ohnolog complement derives from chromosome 6H alone and a further 33% is found on chromosomes 3H and 7H together, leaving just 17% between the other four chromosomes. These biases derive mainly from the fact that the large majority of ohnolog pairs belongs to four WGD-derived regions shared by chromosomes 1H and 3H, 2H and 6H and 6H and 7H (Figure 3). Almost all of chromosomes 6H and 7H, together with the long arms of chromosomes 1H, 2H and 3H retain ohnologs. All the ohnologous regions combined correspond to genomic space containing 46% of mapped barley genes (16 013/35 134), or 51% of the barley physical map (1.98 Gbp of the total 3.90 Gbp). Thus, roughly half of the barley genome lacks detectable duplicated gene pairs derived from the WGD.

We also scrutinized the sequence evolution of the 290 ohnolog pairs. Average pairwise nucleotide identity between them is 69.8% and the average Ks (synonymous substitution rate) is 1.290, consistent with a non-coding nucleotide substitution rate of 1.38 × 10^−9^ substitutions/site/year, assuming a divergence time of 60 MYa. Ka, Ks and Ka/Ks values are all slightly higher between HR pairs (0.18, 1.30, 0.17) as compared with LR–HR pairs (0.17, 1.27, 0.15) but the differences are not significant. The frequency distributions for the three evolutionary parameters (Figure S11) show slight skewing of HR–HR pairs towards higher Ka/Ks values. This corresponds to 25 ohnolog pairs with Ka/Ks > 0.3, 21 of which are HR.

We next explored the functional evolution of the barley ohnolog pairs. GO term enrichment analysis of the 290 pairs (Du *et al*., 2010) showed significant over-representation in several biochemical pathways or functions, particularly intracellular signaling and phosphate modification (Table S4). Thus, there is evidence that barley ohnolog pairs have been preserved because of the functions of their gene products.

Finally, we investigated the gene expression of the ohnolog pairs, using RNA-seq data from the IBGSC dataset (IBGSC, 2012). Analysis of covariance testing revealed no significant difference in differential gene expression between HR–HR and LR–HR pairs (68 and 71% respectively). Similarly, K-means cluster analysis of the same data set showed no evidence for clustering by ohnolog pair type. We conclude that retained ohnolog pairs show no significant effect of the LR-PC region upon their expression divergence. We also looked for bias in ohnolog expression by chromosomal region. Of the 290 ohnolog pairs, 180 show significant bias in averaged RNA level within gene pairs. When these are assigned to ohnolog region (Table S5), no evidence of regional bias in ohnolog expression is evident (χ^2^ = 2.85; *P* = 0.83).

### The LR-PC region and local gene duplication

Local gene duplication is the result of unequal exchange during recombination, so reduced recombination should lead to reduction in locally duplicated gene families (Zhang and Gaut, 2003; Rizzon *et al*., 2006). Local duplications appear as points on the diagonal in a second-best blast plot of a chromosome to itself (e.g. Figure S6). When our mapped gene dataset was analysed 7379 locally duplicated genes were identified, or 21% of the total mapped gene complement. 1297 (17.6%) of these locally duplicated genes reside in the PC region, compared to 24.7% of all genes (Table 2). Therefore the barley PC region is depleted by roughly 29% for locally duplicated genes, relative to the non-PC region. Both this difference and all corresponding differences for the seven barley chromosomes are significant (Table 2).

**Table 2:** Distribution of locally duplicated genes by genome compartment and chromosome

Chromosome	Locally duplicated genes				All mapped genes				Ratio of percentage^a^	*P*-value^b^
	HR	LR	Total	% LR	HR	LR	Total	% LR		
1H	702	150	852	17.6	3405	895	4300	20.8	0.85	0.0374
2H	937	200	1137	17.6	4183	1398	5581	25.0	0.70	<0.0001
3H	1055	229	1284	17.8	4172	1384	5556	24.9	0.72	<0.0001
4H	480	169	649	26.0	2421	1226	3647	33.6	0.77	0.0002
5H	1120	168	1288	13.0	4711	1148	5859	19.6	0.67	<0.0001
6H	676	217	893	24.3	2820	1487	4307	34.5	0.70	<0.0001
7H	1112	164	1276	12.9	4733	1150	5883	19.5	0.66	<0.0001
Total	6082	1297	7379	17.6	26445	8688	35133	24.7	0.71	<0.0001
%	82.4	17.6	100	–	75.3	24.7	100	–	–	–

a % LR (locally duplicated genes)/% LR (all mapped genes).

b Two-tailed *P-*value for chi-squared test with Yate's correction.

To investigate the distribution of local gene duplications in the barley genome, we plotted the densities of local gene duplications across all chromosomes (Figure S12). No strong trend was seen, but there is a slight tendency for increased local gene duplication density towards the telomeres. Analysis of gene ontology of local duplications using AgriGO v1.2 (Du *et al*., 2010) revealed no specific GO terms to be enriched in the local duplication dataset compared to all genes.

## Discussion

### The LR-PC region of barley is permissive for gene expression

The LR-PC region of barley contains roughly a quarter of the genes for the species and at least 48% of the sequenced barley genome in an environment where genetic recombination is suppressed at least 20-fold relative to the average rate and chromatin remains largely compacted during interphase. The exact DNA sequence of this region remains somewhat unclear at present, because it is extremely difficult to sequence through the high density of nested repeats therein. Nevertheless, it is highly likely that the great majority of genes in the LR-PC region are embedded within extensive TE clusters, which are known to be functionally repressed in plants via the feedback loop of RNA silencing and methylation of DNA and histones (Lippman *et al*., 2004; Hall *et al*., 2012). Despite these constraints, our results show that both average mRNA level and developmental transcriptional specificity for the genes of the LR-PC region are indistinguishable from those for the HR gene compartment. The LR-PC region of barley is therefore wholly permissive for gene expression, implying that the genes within it are as accessible to the transcription machinery as are corresponding HR genes.

This conclusion contrasts somewhat with the perceived situation for rice (Yin *et al*., 2008; Wu *et al*., 2011) and soybean (Du *et al*., 2012) but these data are also consistent with reasonably abundant gene expression within the LR-PC region of angiosperm plants. For Arabidopsis, genes within heterochromatin are expressed at comparable levels to their euchromatic counterparts and carry local chromatin signatures such as DNA methylation and histone methylation, which are characteristic of euchromatic genes (Lippman *et al*., 2004). The barley PC region is far more dense in TEs than Arabidopsis and the barley HR region also carries high TE densities, but this environment seems to have little or no effect upon gene expression, so we expect that local chromatin structure in the two genomic compartments will turn out to be similar.

### Restricted recombination of the LR-PC region affects overall nucleotide diversity and selection in wild barley

Our data show that the low recombination rate within the PC region of barley has acted upon the genes within it to constrain gene diversity, in agreement with previous studies (IBGSC, 2012). This phenomenon is widespread in nature and has been ascribed to a combination of selective sweeps via fixation of advantageous allele variants and background selection against deleterious mutations (Hudson, 1994; Wright *et al*., 2006). It should be noted that selective sweeps are not confined to LR regions, it is just that their extent is larger there (Begun and Aquadro, 1992). This fits with our data that show clear trends in both recombination rate and diversity to increase towards the telomeres (Figure S3) of most barley chromosomes.

Recombination allows selection to act upon genes, instead of large genomic regions and the LR-PC region of barley shows a 20-fold restricted recombination rate relative to the average for the species. Our data show that this reduction is associated with higher π_a_/π_s_ ratios for LR-PC region genes over their HR counterparts, which is consistent with Hill–Robertson effects. This build-up in poorly selected, protein-altering polymorphism is a genetic burden for the species and begs the question how such a large fraction of the barley genome has become involved. We suggest that the highly diverse and successful retrotransposon population in this lineage has played a major role in the expansion of its LR-PC region. Barley and the other Triticeae species have much larger genomes than their relatives such as *Brachypodium* and rice, most of the extra DNA is retrotransposons and most of these reside in the LR-PC regions. TEs drive the formation of heterochromatin via the RNAi pathway and recombination is associated with open chromatin (Berchowitz *et al*., 2009).

The potential impact of genetic bottlenecking and inefficient purifying selection in 25% of the barley gene complement upon crop performance is difficult to assess but may be considerable. Furthermore, the many loci within the barley LR-PC region that are important for crop improvement are trapped in extended haplotypes which are extremely difficult to break down by genetic crossing to achieve crop improvement. One promising solution to these problems is provided by the LR-PC regions of wild barley, which have considerably more diversity, both genic and haplotype, than the cultivated gene pool and should be considered as potential sources of new diversity for crop improvement in barley and the other economically important Triticeae crops.

### Ohnolog evolution and the LR-PC region

To explore possible long-term effects of the LR-PC region on gene and genome evolution we have collected ohnolog gene pairs derived from the cereal WGD. Our assignment of 290 ohnolog pairs is likely to be an underestimate for two reasons. First, we have used very stringent criteria for defining ohnolog pairs, because false positives might distort the deductions derived from the small number of surviving gene pairs in barley, whereas underestimation of the numbers would be unlikely to greatly affect the broad conclusions. Second, we have only scrutinized the 60% of the total HC barley gene set that is mapped to date. It is therefore likely that the ohnolog pair number will increase significantly, but we think it very unlikely that it will approach the number for rice, which has had 2246 WGD ohnolog pairs defined (Thiel *et al*., 2009). Even if the ohnolog pair number for barley doubles, it still only represents a few percent of the gene complement for the species, indicating that the rate of gene synteny loss has been particularly high in the barley lineage compared with rice. It may not be a coincidence that barley has experienced much higher levels of both segmental rearrangement and gene translocations than rice (Salse *et al*., 2008; Thiel *et al*., 2009; Wicker *et al*., 2011).

It is also clear that ohnolog pair loss has been strongly biased by genomic position, with two chromosomes (6H and 7H) containing ohnologs across more or less their entire extent and the rest showing large gaps in ohnolog-containing regions. We estimate that roughly half of the barley genome, comprising entire short arms of chromosomes 1H, 3H, 4H and 5H plus large regions of 2HS, 4HL and 5HL, retains no evidence for the WGD (Figure 4). It will be interesting to compare the gene content of such regions with homeologous regions in other cereals that retain ohnolog pairs, to discover how this happened. It is important to note that loss of ohnolog pairs does not necessarily mean gene loss. Gene movement has been widespread for both barley and wheat (Wicker *et al*., 2010, 2011).

### Local gene duplication in the barley LR region

We see a significant reduction in local duplication of genes in the LR-PC region and flanking LR regions, relative to the rest of the genome. This was expected, since homologous recombination must occur for tandem duplications to arise and a similar effect has been seen for Arabidopsis and rice (Zhang and Gaut, 2003; Rizzon *et al*., 2006). The LR-PC region is far more extensive for barley than it is for rice and Arabidopsis, because of its larger genome and much greater complement of repetitious DNA. We have therefore been able to map local gene duplication more accurately but we can still only just see this effect (Figure S12). This is consistent with the modest overall reduction that we see in local gene duplication of 29% and it contrasts strongly with >95% reduction on recombination rate. Thus, greatly reduced recombination does not mean greatly reduced gene duplication for barley. We conclude that selection acts to buffer this presumptive dramatic difference in the incidence of local gene duplicates across the genome, preserving rare duplicates in the LR regions and/or eliminating disadvantageous duplicates in the HR genome compartment.

### Effects of chromosomal environment on divergent gene expression

Following WGD events gene loss (genome fractionation) is rapid and biased by genomic region. For maize, gene expression is the most important factor for gene retention (Schnable *et al*., 2011). We find no evidence for genomically-biased fractionation in barley based on gene expression (Table S5). This apparent contradiction may be explained by the fact that the maize WGD event occurred 5–12* *MYa and we are looking at ohnolog pairs that have survived ca. 60 MYa of selection; Thus any bias may have disappeared over this much longer time interval. Another possibility is that the relatively small number of surviving ohnolog pairs in barley represent rare exceptions to biased fractionation. Nevertheless, soybean underwent a WGD around the same time as maize but shows no significant difference between expression level of LR-PC region genes and their HR ohnologs (Gent *et al*., 2012), consistent with our observation.

### Extinction of ohnolog pairs from the LR-PC region

The complete absence of LR–LR ohnolog pairs for barley was perhaps the biggest surprise from our studies. We therefore looked in published plant genome data for sorghum (Paterson *et al*., 2009), rice (Rizzon *et al*., 2006; Thiel *et al*., 2009), *Oryza brachyantha* (Chen *et al*., 2013) and maize (Schnable *et al*., 2009). All these species appear to share this property. We therefore performed complete ohnolog analysis on the sequenced genomes of *Brachypodium* and rice, using the same recombination-based criterion for LR regions and the available genetic linkage maps and genome data (Tian *et al*., 2009; Huo *et al*., 2011). These species also show no evidence for LR–LR pairs (Tables S6–S8), showing that LR–LR pairs are at least very rare and perhaps absent from cereal genomes.

To our knowledge the only sequenced plant genome reported to contain LR–LR ohnolog pairs is soybean and all of these pairs are located at LR–HR boundaries (Du *et al*., 2012). These regions may have become LR relatively recently and ohnolog elimination has not yet been completed or they may not be fully within the LR-PC region. We define the LR-PC region as a continuous region with at least 20-fold lower average recombination rate than the genomic average and none of the soybean regions fulfil that criterion, with reduced recombination ratios of between 4-fold and 13-fold (Du *et al*., 2012).

Why are LR–LR ohnolog pairs so rare? Barley's evolutionary lineage has experienced a high level of ohnolog pair loss and even a slight bias towards elimination of LR–LR ohnolog pairs could lead to their extinction. However, this argument is less persuasive for maize and soybean which retain high proportions of ohnolog pairs from more recent WGDs. We therefore suggest that LR–LR ohnolog pairs in plants are eliminated rapidly because neither copy can escape from the repressive environment for gene evolution in the LR-PC region, thus neofunctionalization is inhibited.

In conclusion, the barley LR-PC region is a permissive environment for the expression of genes but it restricts gene evolution and local duplication. It may be that the extinction of LR–LR WGD ohnolog pairs for barley and other plant genomes is a consequence of these restrictions. It is intriguing that these species thrive despite these restrictions on large fractions of their genes.

## Experimental procedures

### Plant materials

Fourteen *Hordeum spontaneum* germplasm samples from the World Barley Diversity Collection (WBDC; Steffenson *et al*., 2007) were selected to maximise both the diversity of chloroplast haplotypes and global genomic diversity, as judged by principal coordinate analysis (Figure S2) of SNP marker data using 1153 Illumina BOPA1 markers (Close *et al*., 2009).

### Definition of LR regions

Genetic map positions for 35 134 mapped barley genes (26 159 HC and 8975 LC) in the Morex-Barke population (Mayer *et al*., 2011) were plotted against corresponding physical positions (Figure S1). LR regions were defined as continuous genomic regions longer than 2% of the corresponding physical chromosome length, with 20-fold lower recombination rate than the average for the corresponding chromosome (Choo, 1998). For *Brachypodium* and rice the same criterion for LR region was applied to linkage maps of Huo *et al*. (2011) and Tian *et al*. (2009), respectively.

### Genomic transcription level analysis

Pseudo-physical map positions for genes on the Affymetrix Barley1 GeneChip were found by BLAST comparison of its array sequence file (http://www.plexdb.org/), with 79 379 HC and non-HC presumptive barley gene sequences, (IBGSC, 2012). Map positions and the corresponding transcriptomics data (Druka *et al*., 2006; Table S9) were plotted in MS Office Excel.

### RNA-seq data acquisition and analysis

Barley seeds were germinated on moistened sterile filter paper in Petri plates at room temperature in the dark. Embryonic tissue (coleoptile, mesocotyl and seminal roots) was dissected and flash-frozen 4 days post-germination. Total RNA was extracted from 200 mg tissue using TriReagent (Sigma, http://www.sigmaaldrich.com/sigma-aldrich/home.html), with additional phenol–chloroform purification. RNAs were quality checked using a Bioanalyzer 2100 RNA 6000 Nano kit (Agilent, http://www.genomics.agilent.com). Sequencing was carried out on an Illumina GAII instrument (separate lanes per sample) with TruSeq RNA (Illumina, http://www.illumina.com) library generation and single-end 75-bp reads.

Raw sequence reads were quality trimmed from both ends using a base quality cut-off of 20. Identical duplicate reads were removed to reduce the false positive SNP discovery rates. Reads were mapped to a consensus set of 22 651 FLcDNAs, obtained by consolidating two studies (Sato *et al*., 2009; Matsumoto *et al*., 2011). Mapping used the Bowtie tool (Langmead *et al*., 2009), allowing one mismatch per read, to all possible mapping locations on the FLcDNA reference. Reads were mapped one sample at a time and resulting mappings were merged to produce a consolidated single mapping for all lines. To facilitate direct comparison of transcript abundances between and within samples, RPKM (reads/kilobase of reference/million reads) values were computed for all transcripts from the combined mappings of all lines.

### Single nuclear polymorphism (SNP) discovery and validation

SNPs were discovered for each sample using custom-written code implemented as a prototype feature in Tablet software (Milne *et al*., 2013). The raw variant data were pre-filtered to remove variant locations caused by sequencing errors, using both a minor allele frequency cut-off of 0.1 and a minimum read count of 3. To validate mapping and SNP discovery, genotype calls from 10 samples with 1713 SNPs were compared against known corresponding SNP genotypes from Illumina SNP genotyping of the same lines. Validation rates averaged 98% across all SNPs and lines.

### π_a_/π_s_ determinations for *H. spontaneum* genes

π_a_/π_s_ ratios were derived from SNP data by implementing a custom Java code for protein-coding sequence identification and SNP effect prediction (i.e. protein-coding or non-coding and synonymous or non-synonymous change) on each cDNA read set and its corresponding reference sequence. The code used the translation engines supplied with the BioJava application programing interface (Prlic *et al*., 2012) and is available upon request from the authors. Protein-coding regions were defined as the longest open reading frames (ORFs) downstream of an ATG codon in the cDNA reference. Sequences upstream and downstream of these regions were defined as 5′ and 3′ UTRs respectively. Next, 100 putative protein-coding sequences identified above were manually checked using the NCBI ORF Finder tool. Each was subjected to BLASTP analysis and homology across the full sequence to proteins in related species was taken as evidence that the ORF was correctly assigned.

### Ohnolog pair acquisition

Barley gene sequences were queried against themselves, using blastn with an initial e-value cut-off of 1. Self-hits were discarded then multiple high scoring pairs were reduced to the single best pair per query gene. The output was trimmed to exclude highly similar gene pairs with bit scores above 8000 and/or 100% nucleotide identity over >200 bp, plus very weakly related pairs with both bit scores <300 and alignment length <500 bp (these parameters were selected after scrutiny of the rice ohnolog pair set). Editing and X–Y plotting of paralog gene pairs used MS Office Excel except where noted. Barley genes in regions of low orthologous *Brachypodium* gene density (>400 *Brachypodium* gene separation) were removed with custom Java code, with subsequent manual clean-up in MS Office Excel. Ohnolog pairs were selected by a combination of visual inspection of chromosome-by-chromosome X–Y plots in MS Office Excel (‘handpicking’) and analysis with MCScanX (Wang *et al*., 2012; see Appendix S1). Shared synteny blocks between barley and *Brachypodium* (Table S10), obtained by plotting best hits between the two species' genomes (Figure S7), were used to order barley genes by *Brachypodium* gene order. To calculate Ka and Ks scores, aligned sequence pairs were analysed by yn00 (Yang, 2007) in the PAML package. To eliminate ohnolog pairs with unacceptably low alignment quality, alignments were inspected at both protein and DNA levels using both Geneious v6.1.6 (Drummond *et al*., 2011) and UniPro UGENE v1.12 (Okonechnikov *et al.,* 2012). Circos 0.64 (Krzywinski *et al*., 2009) was used to plot the physical locations of the ohnolog pairs. SPSS v21 (IBM Corp., 2012) was used to perform *t*-tests on the π_a_, π_s_ and π_a_/π_s_ data.

The final set of HC ohnolog pairs were assigned corresponding genomic environments (LR or HR), depending upon genetic map location (Table S3). Analysis of gene expression of the ohnologs was performed with 262 pairs with RNA-seq data for eight different tissues (IBGSC, 2012). Ohnologs with AK designations were converted to their MLOC equivalents as RNA-seq data is only available for MLOCs. RNA-seq data given in FPKM values were analysed with a univariate analysis of variance test in SPSS v21. PAST software (Hammer *et al*., 2001) was also used to perform a K-means clustering analysis, followed by a chi-squared test in MS Office Excel.

### Barley local gene duplicate acquisition

All barley genes were blasted against themselves. All second-best hits to genes on the same chromosome as the query were selected (i.e. ignoring gene hits to self). From this dataset of barley best hits, locally duplicated genes were selected by removing hits on the same chromosome that were remote from query genes by >2% of the corresponding chromosome length (these were designated intrachromosomal gene translocations).

### Gene ontology analysis

Putative protein sequences were queried against the NBCI non-redundant protein sequence database, using blastp with default settings. Results were processed in Blast2GO (B2G4Pipe Version 2.5.0) (Conesa *et al*., 2005). Blast2GO takes blast results and assigns GOSlim terms to query sequences, based on GO terms of hit sequences. 96315 GOSlim terms were assigned to 22 465 barley genes. AgriGO version 1.2 (Du *et al*., 2010) was used to separately analyse Gene Ontology (GO) term enrichment for both the ohnolog data set and the tandem gene data set.

### Accession numbers

RNA-seq data for the 14 wild barley samples in this article are deposited in the European Nucleotide Archive (http://www.ebi.ac.uk/ena/) under study accession PRJEB4947 and sample accession numbers ERS369216- ERS369229 for barley samples WBDC016, WBDC032, WBDC035, WBDC115, WBDC142, WBDC170, WBDC173, WBDC182, WBDC227, WBDC255, WBDC307, WBDC319, WBDC336 and WBDC344 respectively.

## Supporting Information

Figure S1 The low recombining peri-centromeric (LR-PC) region of barley. **Figure S2.** Selection of highly diverse *H. spontaneum* lines from the World Barley Diversity Collection by principal coordinate analysis of high throughput SNP marker data. **Figure S3.** Diversity and recombination statistics for barley chromosomes. **Figure S4.** Developmental and tissue-specific RNA expression levels are independent of LR-PC region residency. **Figure S5.** π_a_ and π_s_ statistics among 14 diverse lines across the *H. spontaneum* genome. **Figure S6.** Identification of WGD-derived paralogous regions in the barley genome by visualization of BLAST data. **Figure S7.** Using synteny conservation between barley and *Brachypodium* to order the barley genome. **Figure S8.** Acquisition of synteny-supported barley genes using *Brachypodium* conserved synteny – Chromosome B3H as an example. **Figure S9.** Barley paralogy plots for genes sharing conserved synteny with *Brachypodium*. **Figure S10.** Selection of high-confidence, WGD-derived barley paralog pairs. **Figure S11.** Nucleotide substitution data for barley ohnologs. **Figure S12.** Local gene duplication densities along barley chromosomes.

Table S1 Genetic map locations and mapped gene contents for LR regions of the barley genome. **Table S2.** *H. spontaneum* gene diversity and selection statistics. **Table S3.** Barley ohnolog gene pairs. **Table S4.** Gene ontology terms enriched in barley ohnologs. **Table S5.** Analysis of ohnolog gene expression bias by ohnolog region. **Table S6.** Distributions of ohnologs and ohnolog pairs for *Brachypodium* and rice by genome compartment. **Table S10.** Shared synteny blocks between *Brachypodium* and barley genomes.

Table S7 *Brachypodium* ohnolog MCScanX data.

Table S8 Rice ohnolog MCScanX data.

Table S9 RNA-seq data, with gene assignations and map positions.

Appendix S1 MCScanX analysis.
