# Supplementary material for: The low-recombining pericentromeric region of barley restricts gene diversity and evolution but not gene expression
Source: Plant J. 2014 Aug 5;79(6):981–92. doi: 10.1111/tpj.12600 (PMC4309411; doi:10.1111/tpj.12600)
Supplement: Figure S1 — The low recombining peri-centromeric (LR-PC) region of barley. Figure S2. Selection of highly diverse H. spontaneum lines from the World Barley Diversity Collection by principal coordinate analysis of high throughput SNP marker data. Figure S3. Diversity and recombination statistics for barley chromosomes. Figure S4. Developmental and tissue-specific RNA expression levels are independent of LR-PC region residency. Figure S5. πa and πs statistics among 14 diverse lines across the H. spontaneum genome. Figure S6. Identification of WGD-derived paralogous regions in the barley genome by visualization of BLAST data. Figure S7. Using synteny conservation between barley and Brachypodium to order the barley genome. Figure S8. Acquisition of synteny-supported barley genes using Brachypodium conserved synteny – Chromosome B3H as an example. Figure S9. Barley paralogy plots for genes sharing conserved synteny with Brachypodium. Figure S10. Selection of high-confidence, WGD-derived barley paralog pairs. Figure S11. Nucleotide substitution data for barley ohnologs. Figure S12. Local gene duplication densities along barley chromosomes. [file tpj0079-0981-sd1.docx]

**Supporting Information Figures**

**
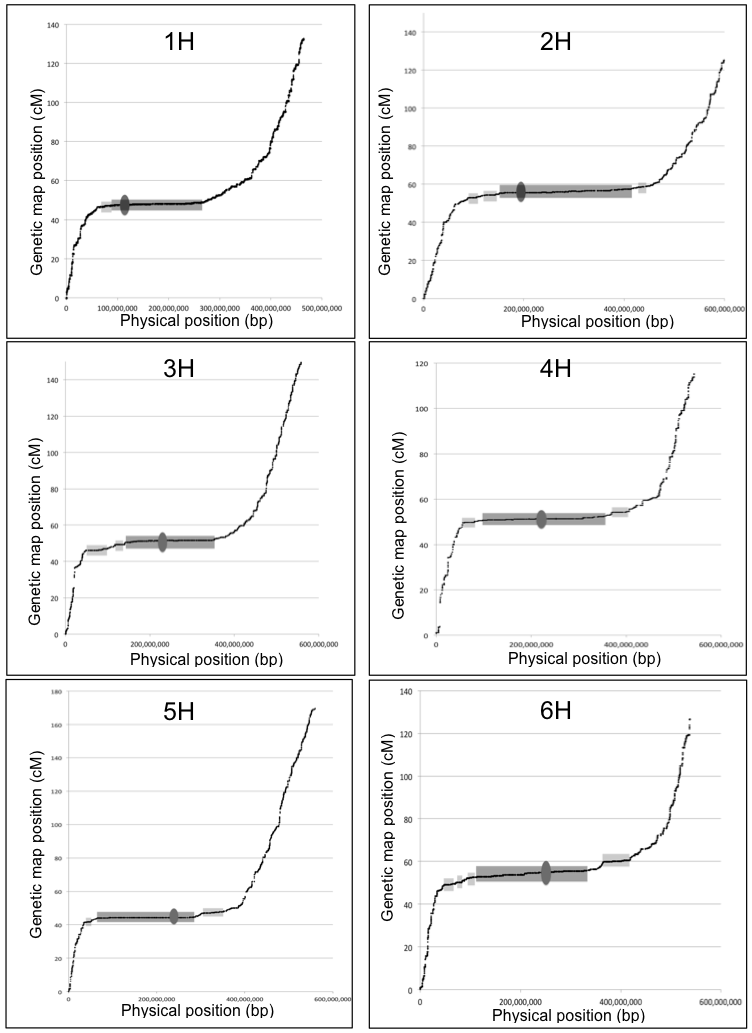
**

see text.

**
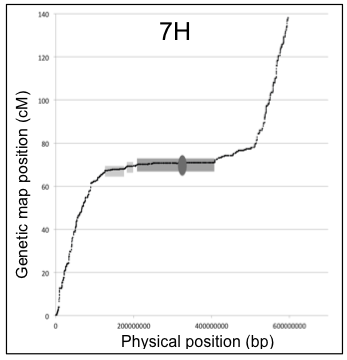
**

**Figure S1. The low recombining peri-centromeric (LR-PC) region of barley**.

Genetic *vs* physical map locations of genes on the seven barley chromosomes. Centromeres (dark grey disks) are surrounded by continuous LR-PC regions (mid-grey bars), with flanking LR regions shown in light grey. For definitions of LR regions see text.


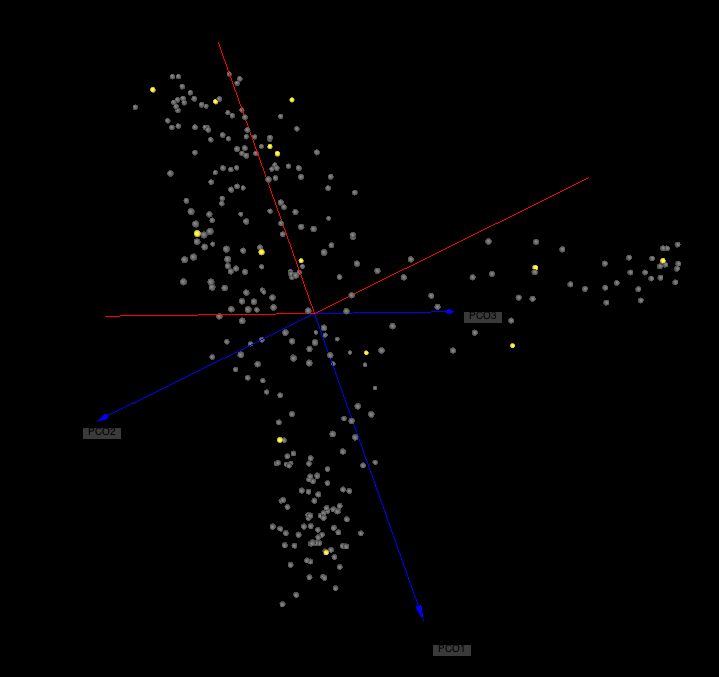


**Figure S2. Selection of highly diverse *H. spontaneum* lines from the World Barley Diversity Collection by principal coordinate analysis of high throughput SNP marker data.**

The 14 selected samples are indicated in yellow. The axes corresponding to the first three principal coordinates are shown.

**
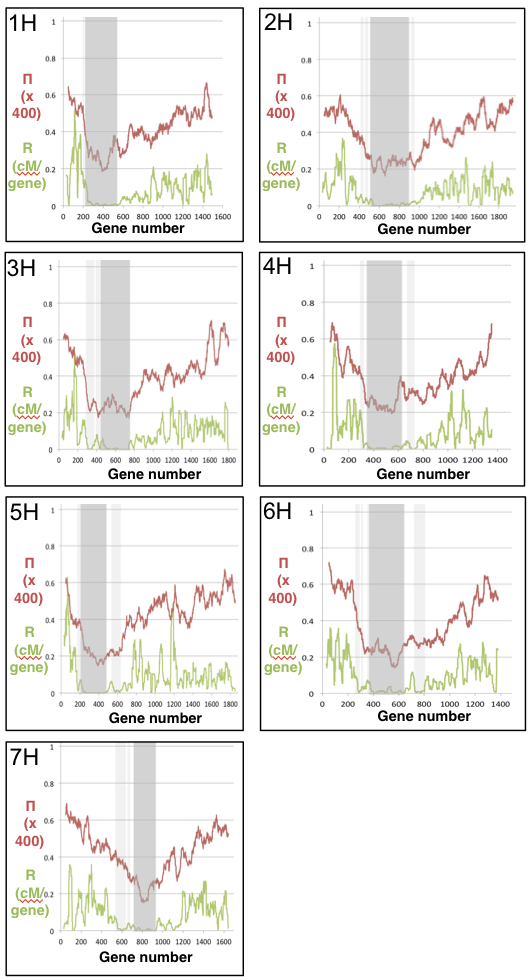
**

**Figure S3: Diversity and recombination statistics for barley chromosomes**.

Rolling averages for gene nucleotide diversity (π, red, 50 genes averaged) and recombination rate (cM/gene interval, green, 30 genes averaged) are plotted against gene order for barley chromosomes 1H-7H. The continuous LR-PC region is shown in mid- grey and flanking LR regions in light grey.


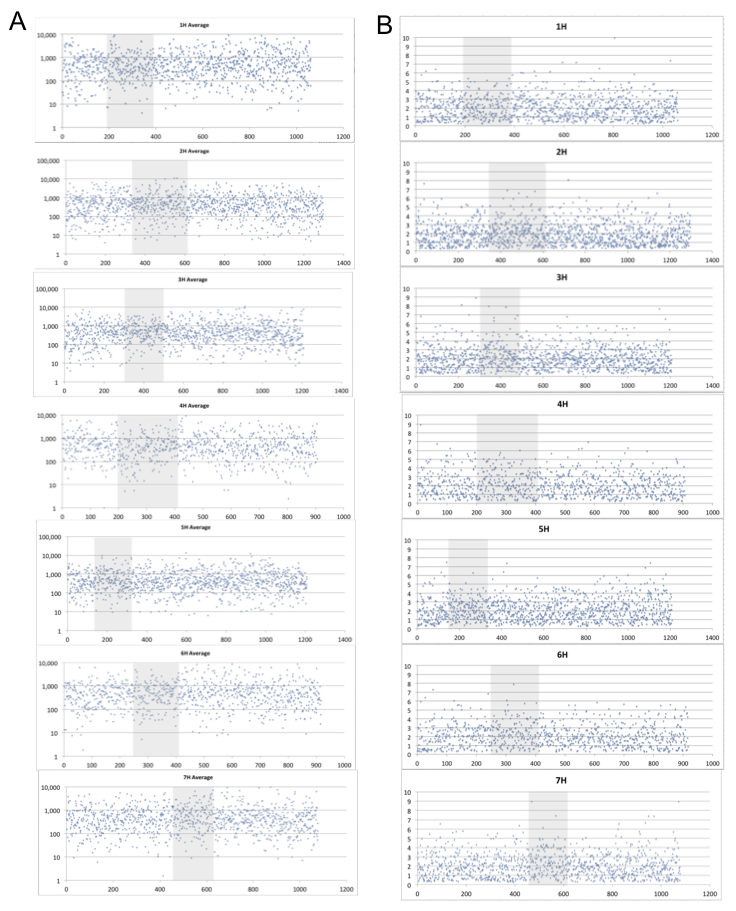


a

b


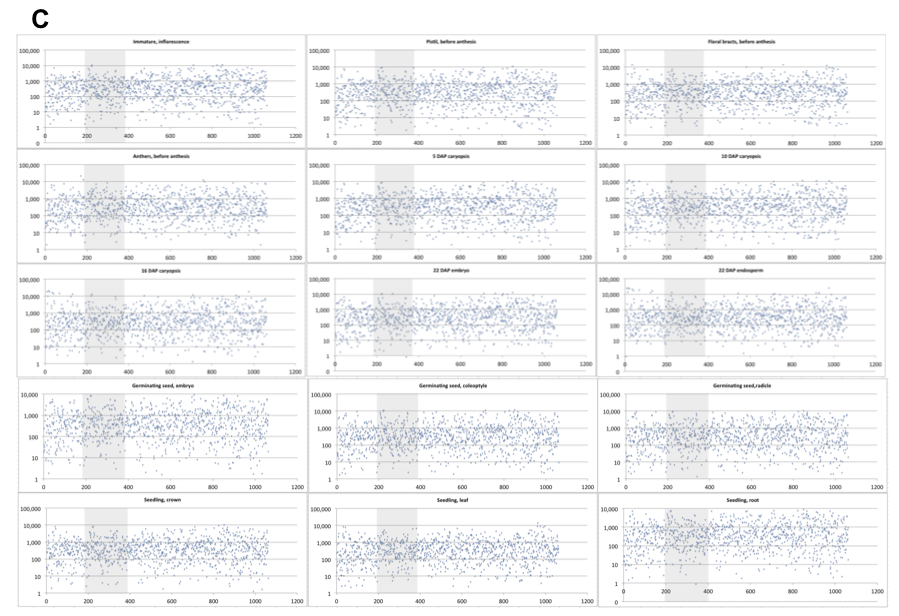


c

**Figure S4. Developmental and tissue-specific RNA expression levels are independent of LR-PC residency.**

RNA levels (arbitrary units) for mapped barley presumptive genes (Druka et al. 2006) are plotted against corresponding linear order on barley chromosomes. The approximate extents of the continuous LR-PC regions are indicated by shading.

1. Average expression levels across chromosomes 1H – 7H respectively, for 15 tissue types, taken from a variety of developmental stages (Druka et al. 2006).
2. Corresponding average developmental and/or tissue specificity quotients (see Results) across chromosomes 1H – 7H respectively.
3. RNA levels across chromosome 1H for each of 15 developmental stages and tissue types.


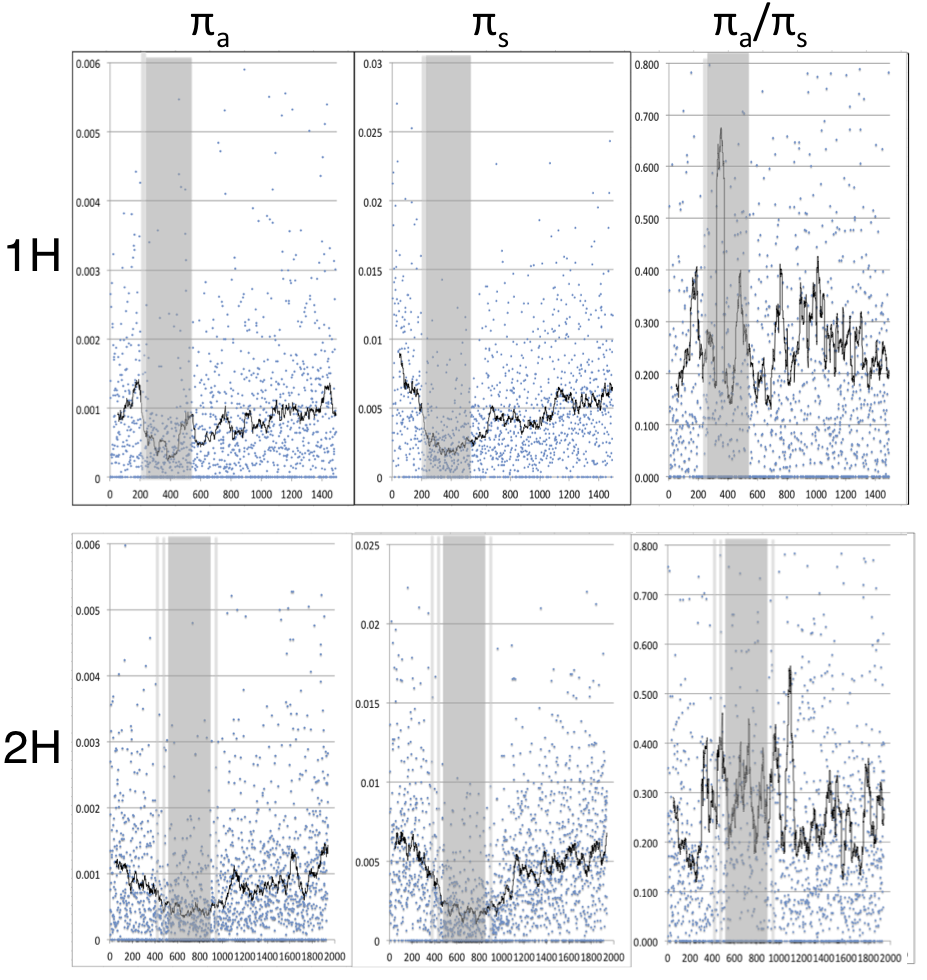


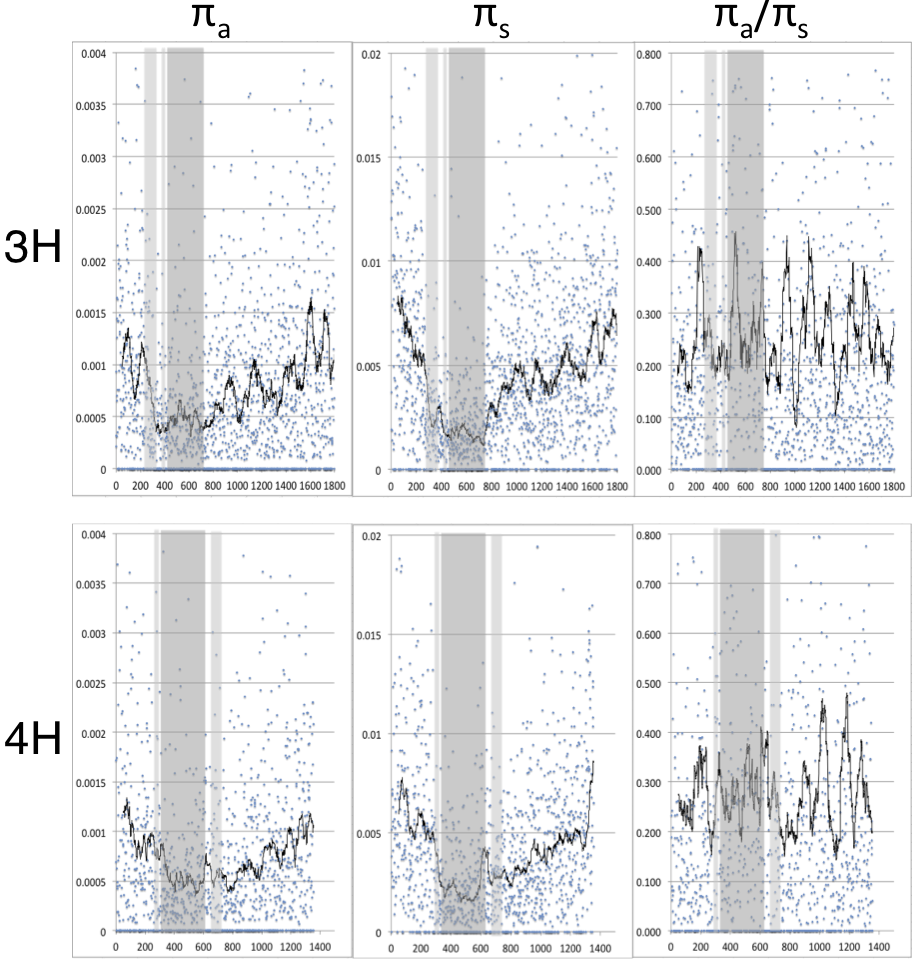


**
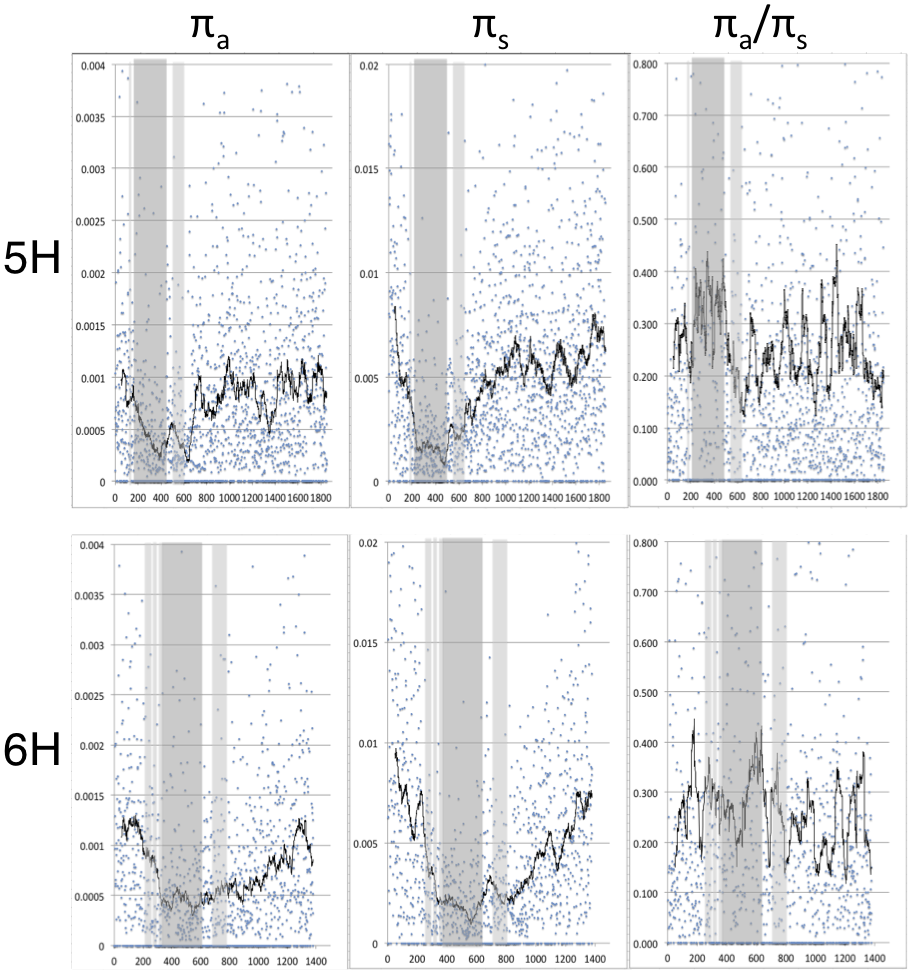
**

**Figure S5. π_a_ and π_s_ statistics among 14 diverse lines across the *H. spontaneum* genome**

π_a_/π_s,_ π_s_ and π_a_ values for barley genes (Y axes) are plotted against their linear order (X axes) on the 7 barley chromosomes. The approximate extents of the LR-PC regions are indicated by shading

**
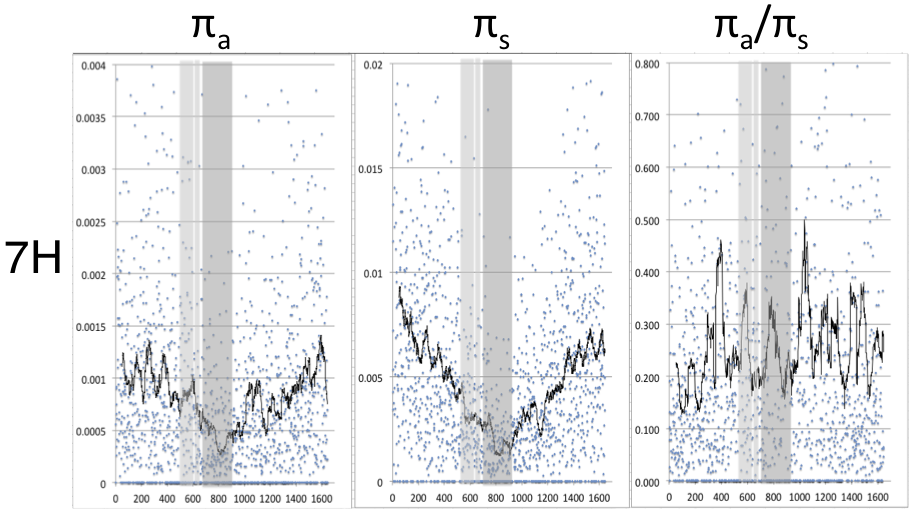
**

**1H 2H 3H 4H 5H 6H 7H**


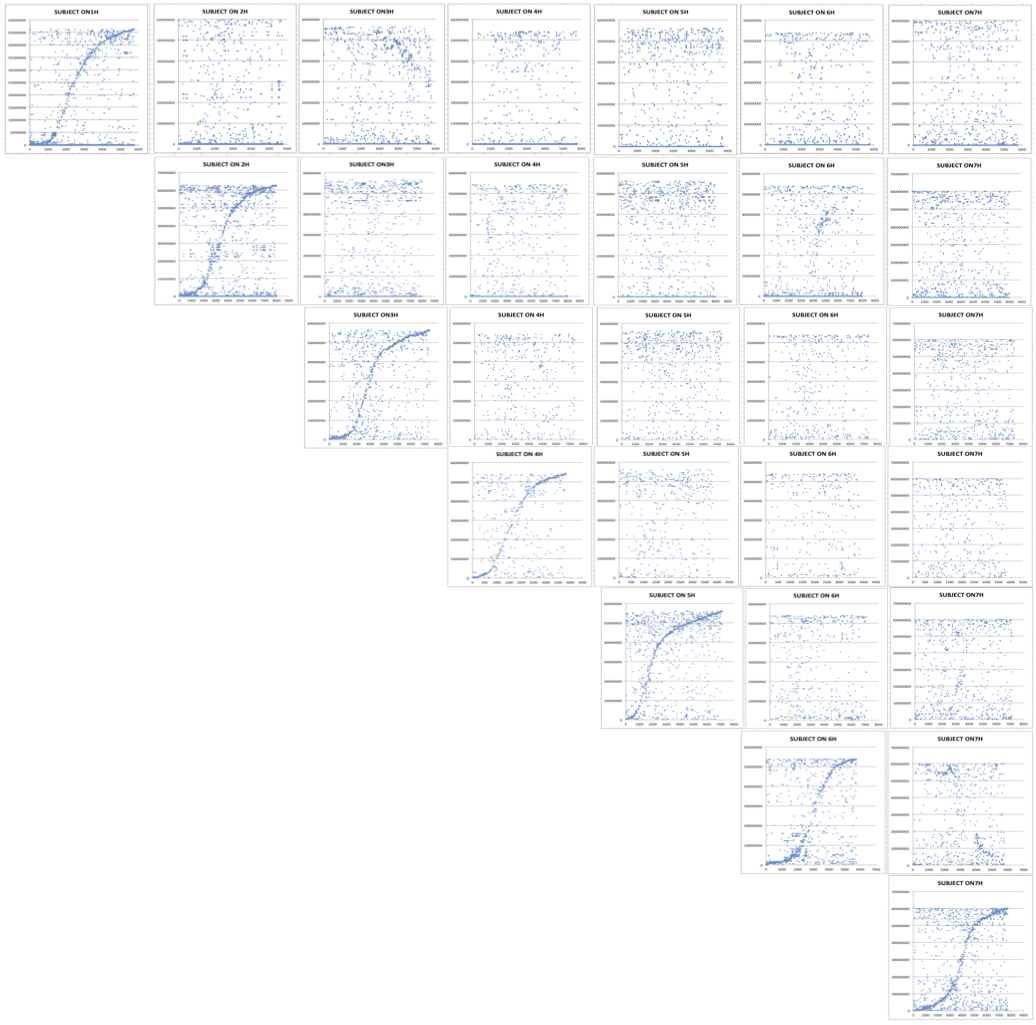


**1H**

**2H**

**3H**

**4H**

**5H**

**6H**

**7H**


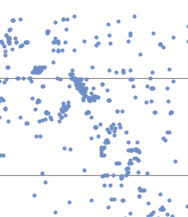


**Figure S6. Identification of WGD-derived paralogous regions in the barley genome by visualization of BLAST data.**

Barley chromosome *vs* barley chromosome BLAST hits. X axes correspond to genetic map order (not cM map position) and Y axes to pseudo-physical map position [International Barley Sequencing Consortium 2012]. Paralogous regions derived from the WGD are arrowed. The inset shows a blow-up of the 1H *vs* 3H arrowed region, to emphasise its complex substructure (see main text).

**
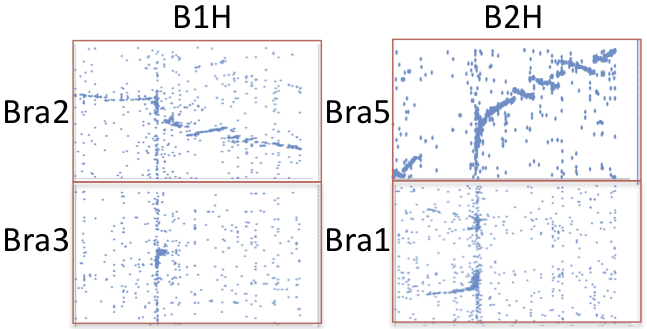

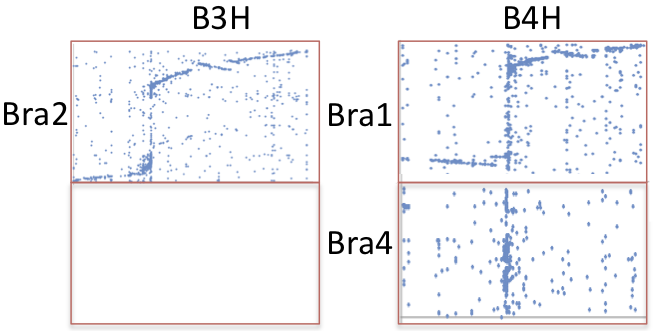

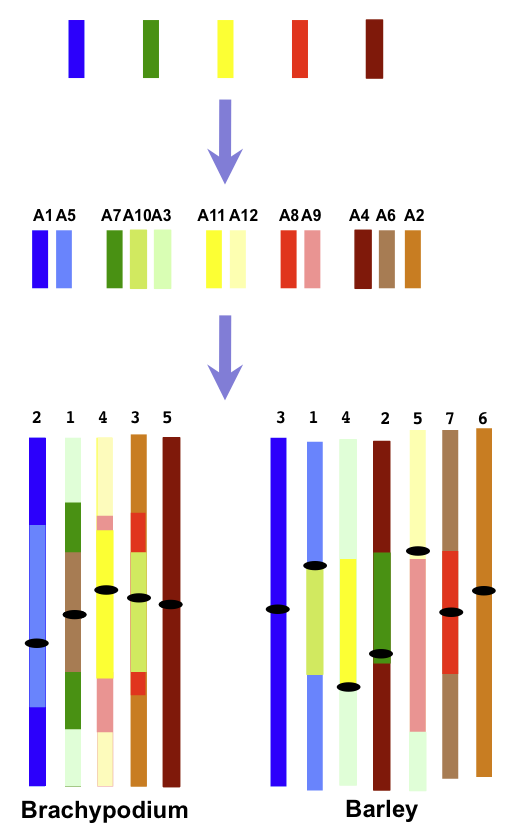
**

**WGD**

**
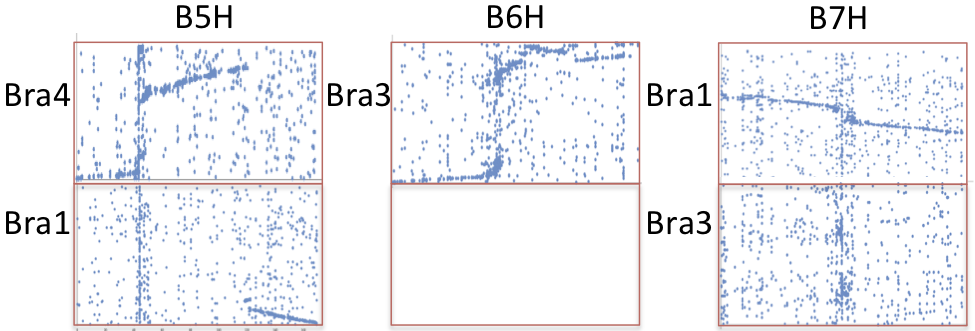
**

**Figure S7. Using synteny conservation between barley and Brachypodium to order the barley genome.**

Barley genetic map order (X-axis) by Brachypodium gene order (Y-axis) plots show the major rearrangements distinguishing the two genomes and their boundaries. The schematic barley genome diagram shows the broad relationships between the genomes of Brachypodium, barley and their proposed 5- and 12-chromosome common ancestors, the latter approximating to the present-day rice genome.


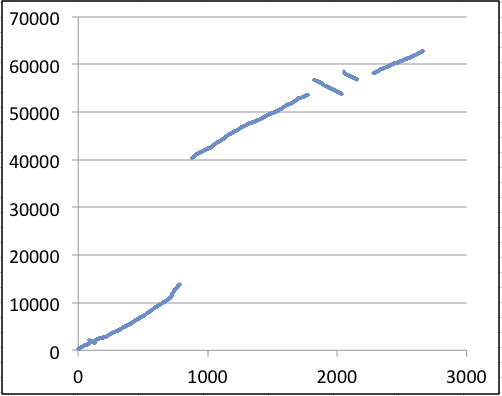

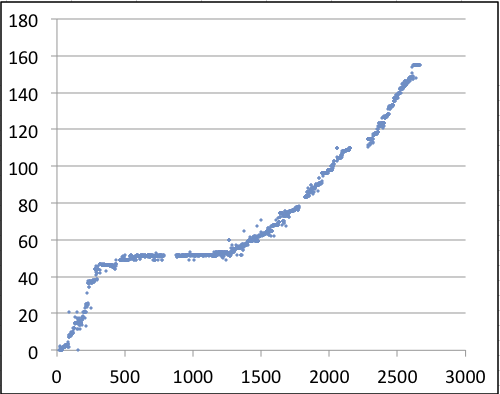
**
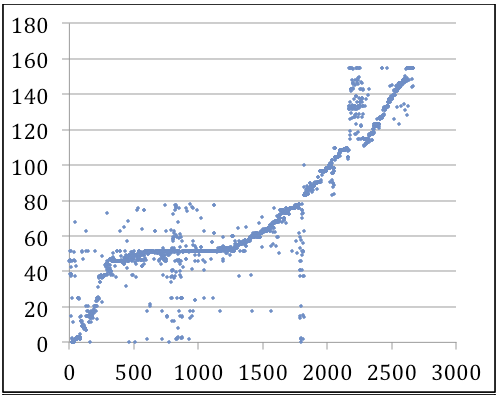
**

**c**

**b**

**a**

**Gene number**

**Map position (cM)**

**Map position (cM)**

**Gene order**

**Gene order**

**Gene order**

**Figure S8. Acquisition of synteny-supported barley genes using Brachypodium conserved synteny – Chromosome B3H as an example**

1. Genetic map positions (Y axis) for barley genes on chromosome 3H with best blastn hits to orthologous Brachypodium chromosome 2, plotted against their corresponding gene order (X axis), after reordering by local Brachypodium gene order.
2. Same plot as **a**, after removal of genes with anomalous barley genetic map positions.
3. The same barley gene set as **b**, plotted by corresponding Brachypodium gene number (Y axis) *vs* Brachypodium gene order (X). The gap at *ca* 800 on the X axis corresponds to the ancestral A5 chromosome introgression in Brachypodium chromosome 2. Two inversions not detected during re-ordering of the barley genome (see Figure S7) appear at position *ca* 2000 on the X axis.

**a**

**1H 2H 3H 4H 5H 6H 7H**


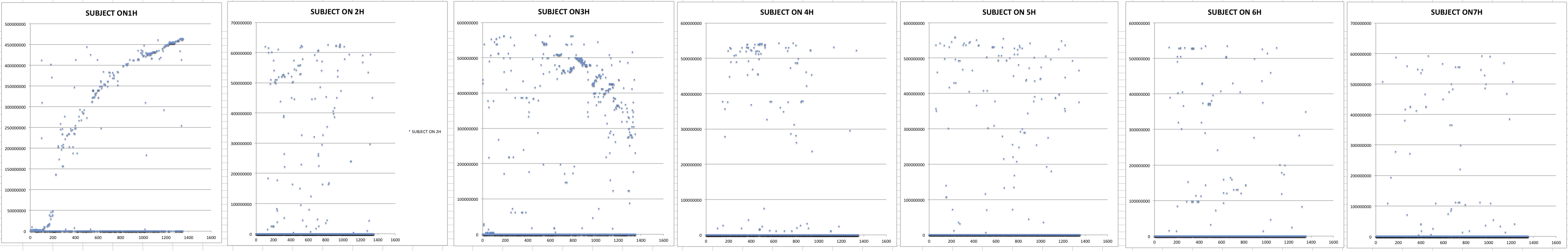

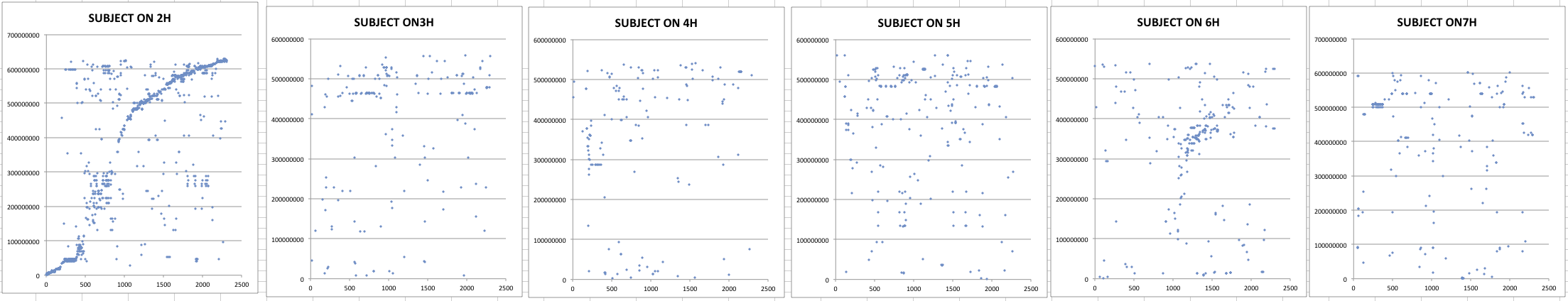

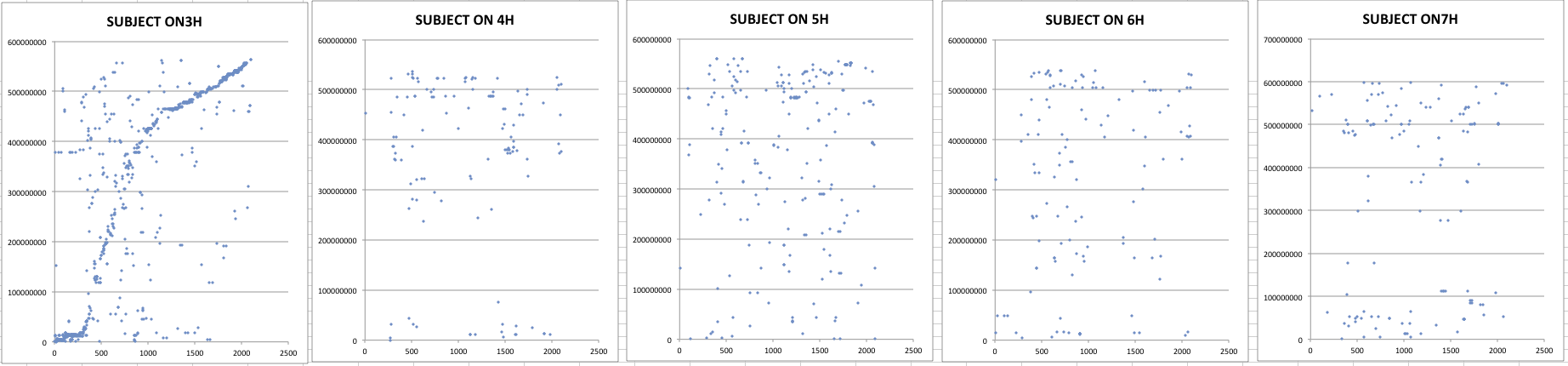

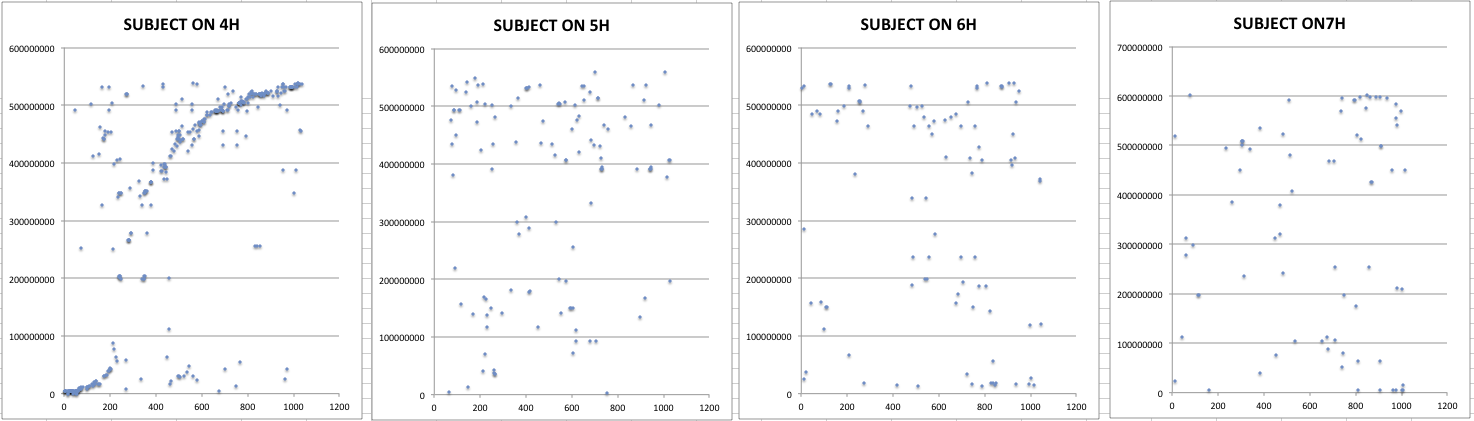

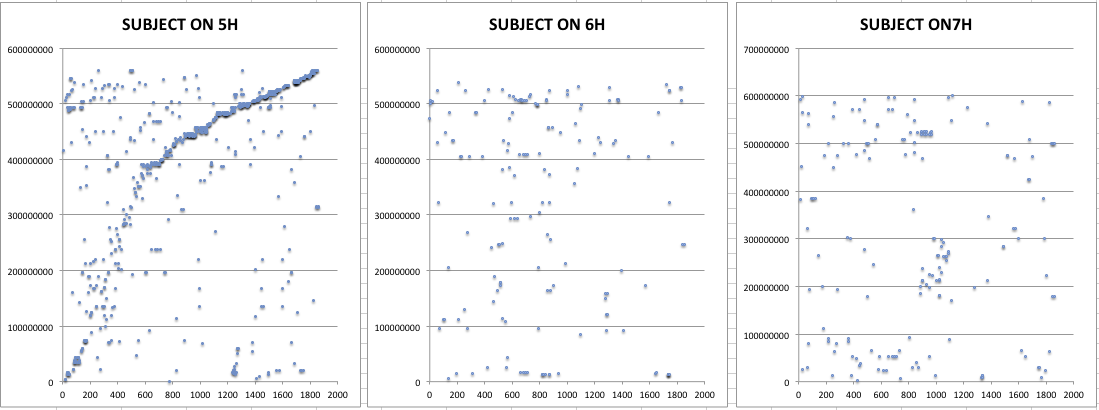

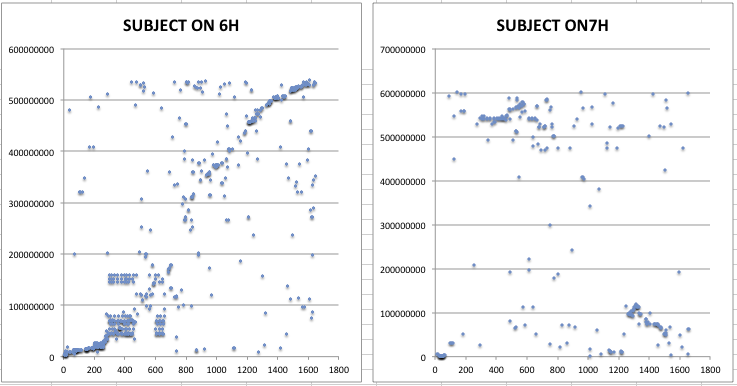

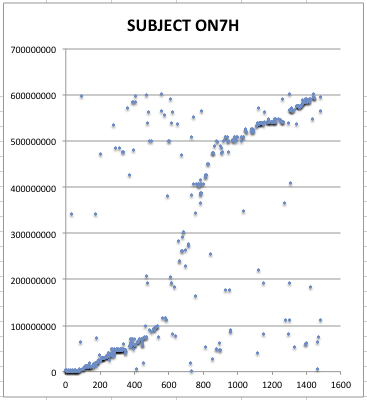


**1H**

**2H**

**3H**

**4H**

**5H**

**6H**

**7H**

**b**

**
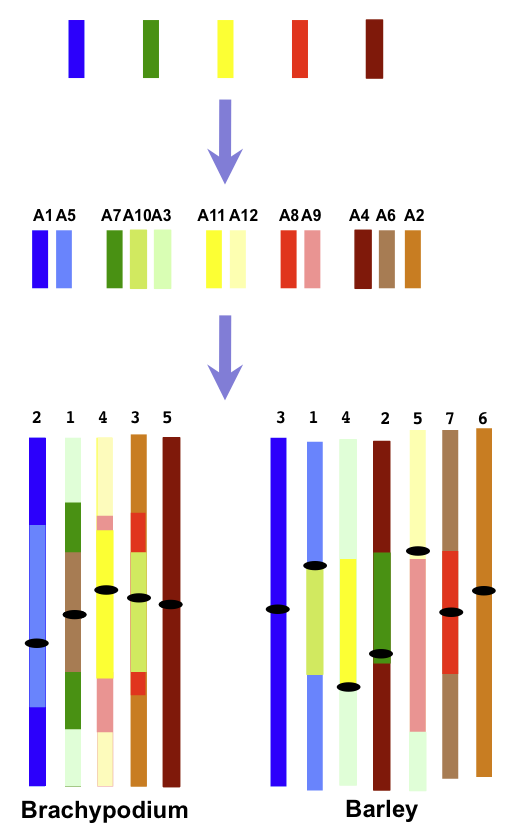
**

**Figure S9: Barley paralogy plots for genes sharing conserved synteny with Brachypodium.**

1. Chromosome by chromosome plots. X axes correspond to reordered barley genome position (see main text) and Y axes to pseudo-physical map position [International Barley Sequencing Consortium 2012]. Seven paralogous regions identified in previous studies (Salse et al. 2008, Thiel et al. 2009) are circled and the regions involved are outlined in the same colours on **b. b.**
2. A schematic of gross chromosomal rearrangements and relationships between the presumtive 12 ancestral cereal chromosomes, their 5 progenitors preceding the WGD and the Brachypodium and barley genomes.

**
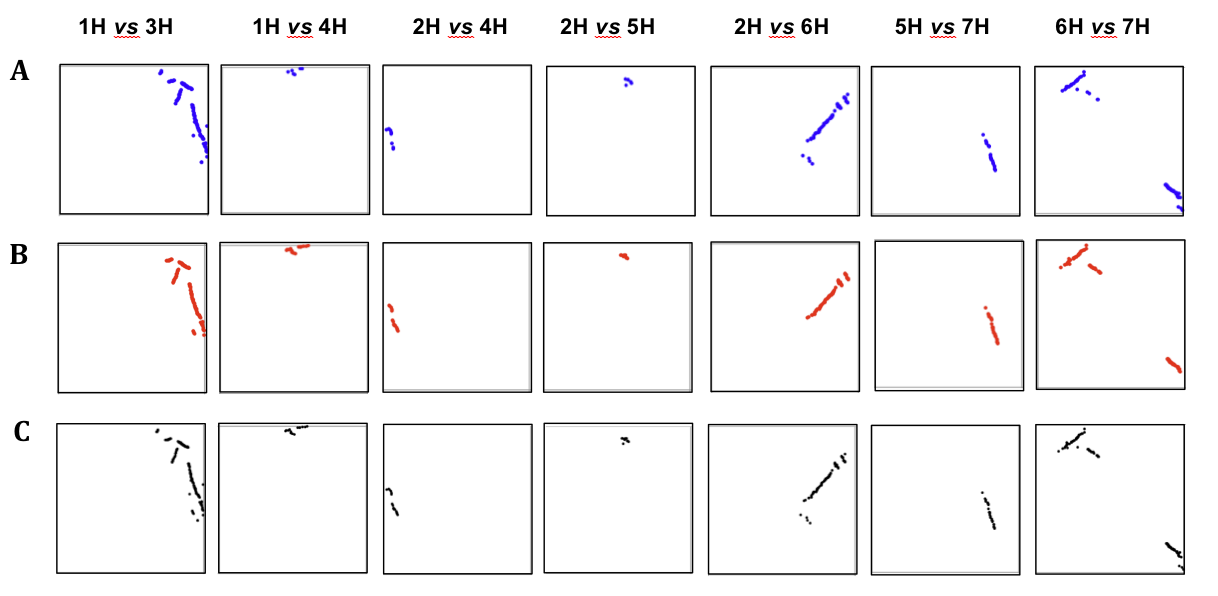
**

a

b

c

**Figure S10. Selection of high-confidence, WGD-derived barley paralog pairs.**

X-Y plots between chromosome pairs (top line) are shown for the 8 ohnologous regions identified in this study. Genes are ordered by pseudo-physical positions of corresponding Brachypodium orthologues (see text) . **a**; Output from manual editing of barley gene pairs from Figure S9A). **b**; Output from MCScanX processing of the same input barley gene set. **c**; Superimposition of plots **a** and **b**.


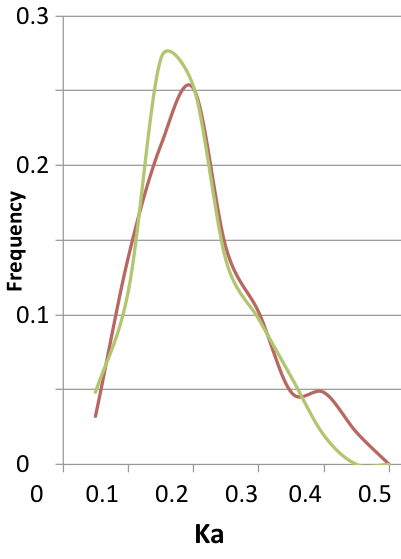

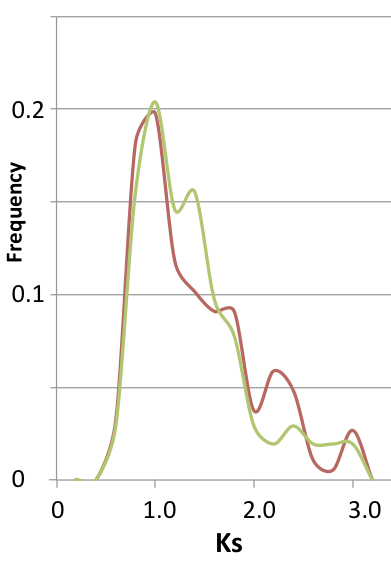

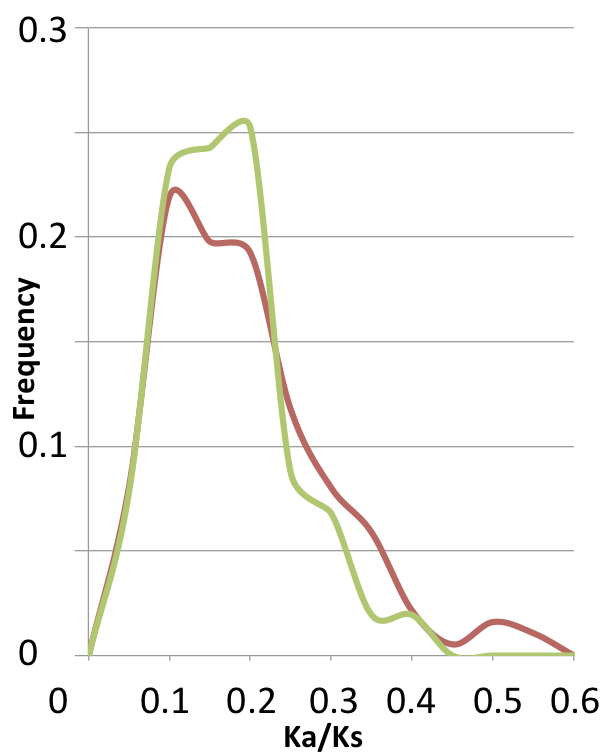


**Figure S11. Nucleotide substitution data for barley ohnologs.**

Frequency distributions of Ka, Ks and Ka/Ks parameters, compared between HR-HR ohnolog pairs (red lines) and LR-HR pairs (green lines).


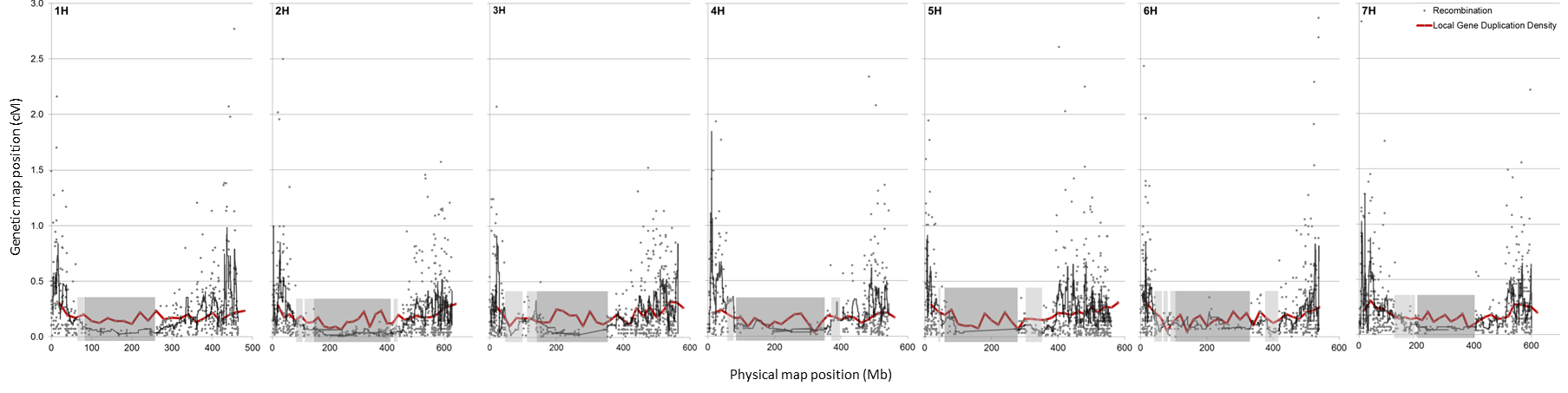


**Figure S12: Local gene duplication densities along barley chromosomes.**

Local gene duplication densities (red lines), calculated as the ratio of number of duplicates to the total number of genes, are plotted over 20 Mb intervals for individual chromsomes. Recombination rates (grey points) represent cM intervals between genes, with rolling averages over 100 genes plotted as black lines. The LR-PC region is shown in dark grey and flanking LR regions in light grey.
