# Supplementary material for: The low-recombining pericentromeric region of barley restricts gene diversity and evolution but not gene expression
Source: Plant J. 2014 Aug 5;79(6):981–92. doi: 10.1111/tpj.12600 (PMC4309411; doi:10.1111/tpj.12600)
Supplement: Table S1 — Genetic map locations and mapped gene contents for LR regions of the barley genome. Table S2. H. spontaneum gene diversity and selection statistics. Table S3. Barley ohnolog gene pairs. Table S4. Gene ontology terms enriched in barley ohnologs. Table S5. Analysis of ohnolog gene expression bias by ohnolog region. Table S6. Distributions of ohnologs and ohnolog pairs for Brachypodium and rice by genome compartment. Table S10. Shared synteny blocks between Brachypodium and barley genomes. [file tpj0079-0981-sd2.docx]

**Table S1: Genetic map locations and mapped gene contents for LR regions of the barley genome**

| Chromosome | Region^a^ | cM start^b^ | cM end^b^ | Number of mapped genes |
| --- | --- | --- | --- | --- |
| 1H | Flanking LR | 46.81 | 47.24 | 87 |
|  | LR-PC | 47.27 | 48.80 | 808 |
| 2H | Flanking LR | 52.20 | 52.90 | 102 |
|  |  | 53.82 | 54.25 | 102 |
|  | LR-PC | 54.69 | 57.44 | 1135 |
|  | Flanking LR | 58.25 | 58.64 | 57 |
| 3H | Flanking LR | 45.22 | 46.88 | 334 |
|  |  | 48.45 | 49.29 | 128 |
|  | LR-PC | 49.65 | 51.77 | 918 |
| 4H | Flanking LR | 49.72 | 49.86 | 106 |
|  | LR-PC | 50.57 | 52.44 | 965 |
|  | Flanking LR | 53.40 | 54.32 | 155 |
| 5H | Flanking LR | 41.81 | 41.81 | 40 |
|  | LR-PC | 43.61 | 44.38 | 821 |
|  | Flanking LR | 46.53 | 48.13 | 286 |
| 6H | Flanking LR | 48.30 | 49.22 | 151 |
|  |  | 49.65 | 50.21 | 96 |
|  |  | 51.56 | 52.55 | 70 |
|  | LR-PC | 52.55 | 55.52 | 914 |
|  | Flanking LR | 59.92 | 60.62 | 260 |
| 7H | Flanking LR | 66.41 | 68.06 | 326 |
|  |  | 68.38 | 69.26 | 100 |
|  | LR-PC | 69.96 | 71.03 | 724 |

^a^ See Figures S1 and S3

^b^ Genetic map positions (Comadran et al. 2012)

**Table S2. *H. spontaneum* gene diversity and selection statistics**

|  | **LR**^a^ | | | **HR**^a^ | | | **LR/HR πa/πs**^b^ |
| --- | --- | --- | --- | --- | --- | --- | --- |
| **Chromosome** | **π_a_** | **π_s_** | **π_a_/π_s_** | **π_a_** | **π_s_** | **π_a_/π_s_** |  |
| **1H** | 0.0006 (0.001) | 0.0023 (0.0025) | 0.246 | 0.0009 (0.001) | 0.005 (0.0044) | 0.172 | 1.401 |
| **2H** | 0.0005 (0.0006) | 0.002 (0.0023) | 0.230 | 0.0009 (0.001) | 0.0048 (0.0041) | 0.186 | 1.209 |
| **3H** | 0.0005 (0.0007) | 0.0019 0.0022() | 0.243 | 0.0009 (0.0011) | 0.0051 (0.0042) | 0.174 | 1.399 |
| **4H** | 0.0005 (0.0008) | 0.0024 (0.0029) | 0.227 | 0.0008 (0.001) | 0.0046 (0.004) | 0.175 | 1.291 |
| **5H** | 0.0004 (0.0005) | 0.0018 (0.002) | 0.217 | 0.0008 (0.001) | 0.0054 (0.0045) | 0.156 | 1.401 |
| **6H** | 0.0005 0.0007() | 0.0021 0.0023) | 0.248 | 0.0008 (0.001) | 0.0052 (0.0047) | 0.161 | 1.484 |
| **7H** | 0.0006 (0.0008) | 0.0024 (0.0029) | 0.234 | 0.0009 (0.001) | 0.0053 (0.0046) | 0.169 | 1.379 |
| **All** | 0.0006 (0.0006) | 0.0021 (0.0025) | 0.235  (0.010) | 0.0009 (0.0010) | 0.0051 (0.0044) | 0.170  (0.011) | 1.382 |

^a^ π gene values are averaged within chromosome compartments.

^b^ π_a_/π_s_ value for LR region, divided by corresponding value for HR region

Standard deviations are in brackets.

**Table S3. Barley ohnolog gene pairs**

| **Query Gene** | | | | | **Hit gene** | | | | | **Ohnolog pair type**^a^ |
| --- | --- | --- | --- | --- | --- | --- | --- | --- | --- | --- |
| **Gene name** | **chr** | **cM** | **Physical map position bp** | **Genomic compartment**^a^ | **Gene name** | **chr** | **cM** | **Physical map position (bp)** | **Genomic compartment**^a^ |  |
| AK369282 | 1 | 48.938 | 263316480 | HR | AK368348 | 4 | 104.582 | 530897320 | HR | HR-HR |
| MLOC_54274.2 | 1 | 50.177 | 276923000 | HR | AK368883 | 4 | 99.079 | 519537680 | HR | HR-HR |
| MLOC_64004.1 | 1 | 49.985 | 276412120 | HR | MLOC_58922.1 | 4 | 97.557 | 513598080 | HR | HR-HR |
| MLOC_75615.1 | 1 | 51.558 | 288571560 | HR | AK353686 | 4 | 97.610 | 513598080 | HR | HR-HR |
| MLOC_53357.1 | 1 | 49.532 | 271623240 | HR | MLOC_58892.3 | 4 | 112.535 | 539469440 | HR | HR-HR |
| MLOC_67926.1 | 1 | 50.850 | 284168320 | HR | AK251931.1 | 4 | 112.535 | 538963520 | HR | HR-HR |
| AK252157.1 | 1 | 50.850 | 284168320 | HR | AK373965 | 4 | 112.960 | 539469440 | HR | HR-HR |
| MLOC_70189.1 | 1 | 51.487 | 287440680 | HR | AK376221 | 4 | 113.739 | 541443520 | HR | HR-HR |
| MLOC_4354.1 | 1 | 52.691 | 297668200 | HR | MLOC_66975.3 | 4 | 112.119 | 536588920 | HR | HR-HR |
| AK250365.1 | 1 | 52.762 | 300354040 | HR | MLOC_43719.2 | 4 | 112.327 | 538963520 | HR | HR-HR |
| MLOC_66120.2 | 1 | 55.412 | 313697680 | HR | MLOC_53215.1 | 3 | 133.144 | 536406640 | HR | HR-HR |
| MLOC_4926.1 | 1 | 57.295 | 323467640 | HR | MLOC_4352.1 | 3 | 137.075 | 541185600 | HR | HR-HR |
| MLOC_62746.1 | 1 | 55.276 | 311889760 | HR | AK356972 | 3 | 137.075 | 541185600 | HR | HR-HR |
| MLOC_60840.1 | 1 | 54.232 | 309919400 | HR | AK356379 | 3 | 139.589 | 544087200 | HR | HR-HR |
| MLOC_61783.1 | 1 | 54.232 | 309919400 | HR | MLOC_4805.1 | 3 | 139.589 | 544891960 | HR | HR-HR |
| AK374154 | 1 | 61.509 | 361019800 | HR | AK375710 | 3 | 108.428 | 508690160 | HR | HR-HR |
| MLOC_61156.1 | 1 | 61.509 | 361019800 | HR | AK373607 | 3 | 108.428 | 508690160 | HR | HR-HR |
| MLOC_61466.1 | 1 | 61.420 | 358608000 | HR | MLOC_57214.1 | 3 | 108.322 | 506688800 | HR | HR-HR |
| MLOC_69621.2 | 1 | 61.154 | 357349400 | HR | MLOC_57215.1 | 3 | 108.322 | 506688800 | HR | HR-HR |
| MLOC_37761.1 | 1 | 60.836 | 350063160 | HR | MLOC_68143.1 | 3 | 108.428 | 506688800 | HR | HR-HR |
| AK362944 | 1 | 61.154 | 357349400 | HR | MLOC_3773.1 | 3 | 108.569 | 508690160 | HR | HR-HR |
| MLOC_63743.1 | 1 | 60.995 | 354380840 | HR | AK362892 | 3 | 109.012 | 510061600 | HR | HR-HR |
| MLOC_44513.2 | 1 | 60.995 | 354380840 | HR | MLOC_59261.1 | 3 | 108.888 | 509294040 | HR | HR-HR |
| MLOC_73207.1 | 1 | 61.154 | 357349400 | HR | AK364124 | 3 | 108.888 | 509294040 | HR | HR-HR |
| AK249744.1 | 1 | 65.658 | 364483120 | HR | AK251447.1 | 3 | 72.344 | 456179880 | HR | HR-HR |
| AK250695.1 | 1 | 67.334 | 373965400 | HR | MLOC_81233.1 | 3 | 73.088 | 457798080 | HR | HR-HR |
| MLOC_53822.1 | 1 | 72.380 | 389364960 | HR | MLOC_6252.1 | 3 | 74.715 | 461934720 | HR | HR-HR |
| MLOC_62765.1 | 1 | 70.892 | 383276560 | HR | AK357864 | 3 | 73.159 | 459524160 | HR | HR-HR |
| AK368252 | 1 | 71.412 | 385054720 | HR | MLOC_19561.1 | 3 | 75.708 | 465497240 | HR | HR-HR |
| MLOC_71567.1 | 1 | 72.592 | 391503960 | HR | MLOC_51655.7 | 3 | 75.956 | 468140920 | HR | HR-HR |
| MLOC_55611.1 | 1 | 72.592 | 391503960 | HR | MLOC_16086.1 | 3 | 76.133 | 470396480 | HR | HR-HR |
| AK366915 | 1 | 72.380 | 389110760 | HR | AK375838 | 3 | 76.062 | 468140920 | HR | HR-HR |
| AK372018 | 1 | 72.082 | 385413080 | HR | AK367133 | 3 | 77.408 | 471528600 | HR | HR-HR |
| MLOC_55931.1 | 1 | 73.123 | 392762560 | HR | AK376811 | 3 | 77.691 | 472433800 | HR | HR-HR |
| AK354185 | 1 | 79.381 | 399745000 | HR | MLOC_44231.1 | 3 | 100.283 | 499671640 | HR | HR-HR |
| AK252730.1 | 1 | 80.241 | 399745000 | HR | AK353914 | 3 | 98.229 | 499289720 | HR | HR-HR |
| AK354090 | 1 | 79.561 | 399745000 | HR | AK371805 | 3 | 98.229 | 499289720 | HR | HR-HR |
| AK355901 | 1 | 80.241 | 399745000 | HR | AK372120 | 3 | 98.229 | 499289720 | HR | HR-HR |
| AK357982 | 1 | 80.241 | 399745000 | HR | MLOC_72428.4 | 3 | 98.229 | 499289720 | HR | HR-HR |
| AK373685 | 1 | 81.524 | 402450680 | HR | AK362505 | 3 | 98.229 | 499289720 | HR | HR-HR |
| AK250937.1 | 1 | 83.569 | 404594640 | HR | AK372762 | 3 | 96.424 | 493200080 | HR | HR-HR |
| AK372000 | 1 | 82.507 | 404594640 | HR | AK361897 | 3 | 96.424 | 492622240 | HR | HR-HR |
| AK362752 | 1 | 79.674 | 399745000 | HR | AK361372 | 3 | 96.601 | 496830800 | HR | HR-HR |
| AK249154.1 | 1 | 82.507 | 403767560 | HR | MLOC_37763.1 | 3 | 96.459 | 496420360 | HR | HR-HR |
| AK366205 | 1 | 86.473 | 407971160 | HR | MLOC_35096.3 | 3 | 96.459 | 494069320 | HR | HR-HR |
| AK248239.1 | 1 | 86.473 | 410870280 | HR | AK360384 | 3 | 90.652 | 489532160 | HR | HR-HR |
| MLOC_70659.2 | 1 | 85.752 | 407518560 | HR | MLOC_9835.2 | 3 | 90.758 | 489532160 | HR | HR-HR |
| MLOC_4800.1 | 1 | 85.752 | 407518560 | HR | AK363838 | 3 | 90.652 | 489532160 | HR | HR-HR |
| MLOC_7958.1 | 1 | 85.752 | 407518560 | HR | AK354719 | 3 | 90.014 | 484621760 | HR | HR-HR |
| AK252195.1 | 1 | 86.473 | 410384200 | HR | AK364902 | 3 | 90.014 | 486616920 | HR | HR-HR |
| AK354659 | 1 | 86.473 | 407971160 | HR | MLOC_2968.2 | 3 | 90.014 | 486616920 | HR | HR-HR |
| MLOC_36786.1 | 1 | 86.473 | 410384200 | HR | MLOC_5326.1 | 3 | 87.960 | 482006600 | HR | HR-HR |
| AK249326.1 | 1 | 86.686 | 412841880 | HR | AK358438 | 3 | 87.394 | 479154600 | HR | HR-HR |
| MLOC_52791.4 | 1 | 88.244 | 415096200 | HR | MLOC_67313.1 | 3 | 87.394 | 480042440 | HR | HR-HR |
| MLOC_52790.1 | 1 | 88.244 | 415096200 | HR | AK353902 | 3 | 87.394 | 480042440 | HR | HR-HR |
| AK370756 | 1 | 86.969 | 412841880 | HR | AK362455 | 3 | 86.367 | 479154600 | HR | HR-HR |
| MLOC_73532.1 | 1 | 86.473 | 410870280 | HR | MLOC_11257.1 | 3 | 87.960 | 481690400 | HR | HR-HR |
| AK377048 | 1 | 86.473 | 407971160 | HR | MLOC_63902.1 | 3 | 84.385 | 477422320 | HR | HR-HR |
| MLOC_66523.5 | 1 | 95.184 | 425396880 | HR | AK374725 | 3 | 67.989 | 448046720 | HR | HR-HR |
| MLOC_62591.1 | 1 | 95.609 | 428338160 | HR | MLOC_63031.2 | 3 | 67.989 | 448046720 | HR | HR-HR |
| AK249327.1 | 1 | 95.609 | 425396880 | HR | MLOC_51142.2 | 3 | 68.201 | 452420200 | HR | HR-HR |
| MLOC_18933.1 | 1 | 95.609 | 425396880 | HR | MLOC_64849.1 | 3 | 68.201 | 450929720 | HR | HR-HR |
| MLOC_68123.3 | 1 | 93.697 | 423306240 | HR | MLOC_65509.2 | 3 | 67.918 | 447466400 | HR | HR-HR |
| AK363253 | 1 | 95.609 | 425396880 | HR | AK362212 | 3 | 64.802 | 441085360 | HR | HR-HR |
| AK374068 | 1 | 95.609 | 425396880 | HR | AK366584 | 3 | 67.635 | 445699400 | HR | HR-HR |
| AK375166 | 1 | 94.618 | 423864240 | HR | AK363019 | 3 | 64.873 | 444693760 | HR | HR-HR |
| AK360842 | 1 | 93.697 | 423306240 | HR | MLOC_64975.2 | 3 | 53.063 | 366675440 | HR | HR-HR |
| AK357487 | 1 | 93.378 | 423306240 | HR | AK354806 | 3 | 62.960 | 435824040 | HR | HR-HR |
| AK376294 | 1 | 93.059 | 421950920 | HR | MLOC_75554.1 | 3 | 62.960 | 435824040 | HR | HR-HR |
| MLOC_55738.1 | 1 | 94.334 | 423639800 | HR | MLOC_81502.2 | 3 | 62.677 | 434379440 | HR | HR-HR |
| MLOC_54908.1 | 1 | 92.493 | 419628400 | HR | MLOC_61686.3 | 3 | 61.898 | 428699000 | HR | HR-HR |
| AK376768 | 1 | 91.147 | 417777080 | HR | MLOC_58725.1 | 3 | 61.898 | 426657960 | HR | HR-HR |
| MLOC_61996.2 | 1 | 90.297 | 416529640 | HR | AK366462 | 3 | 61.898 | 429133000 | HR | HR-HR |
| AK369582 | 1 | 92.493 | 419628400 | HR | MLOC_64503.1 | 3 | 59.632 | 420906840 | HR | HR-HR |
| AK369841 | 1 | 90.297 | 416529640 | HR | MLOC_68083.6 | 3 | 59.632 | 420906840 | HR | HR-HR |
| MLOC_15477.1 | 1 | 95.609 | 428338160 | HR | MLOC_19742.1 | 3 | 59.278 | 411717200 | HR | HR-HR |
| AK250931.1 | 1 | 104.993 | 436546960 | HR | MLOC_76894.1 | 3 | 61.650 | 426113600 | HR | HR-HR |
| AK365929 | 1 | 103.824 | 435738480 | HR | MLOC_74647.1 | 3 | 61.898 | 426657960 | HR | HR-HR |
| AK368020 | 1 | 103.824 | 434515840 | HR | AK363457 | 3 | 60.836 | 425819720 | HR | HR-HR |
| MLOC_32433.1 | 1 | 103.824 | 435738480 | HR | MLOC_54950.1 | 3 | 61.827 | 426657960 | HR | HR-HR |
| AK369934 | 1 | 103.824 | 435738480 | HR | MLOC_13243.1 | 3 | 59.632 | 417066560 | HR | HR-HR |
| AK358521 | 1 | 101.062 | 434515840 | HR | MLOC_74707.2 | 3 | 59.136 | 411717200 | HR | HR-HR |
| MLOC_65986.1 | 1 | 101.062 | 434515840 | HR | MLOC_51359.1 | 3 | 59.136 | 411717200 | HR | HR-HR |
| AK368951 | 1 | 100.921 | 432422720 | HR | MLOC_56148.2 | 3 | 57.542 | 407217240 | HR | HR-HR |
| MLOC_61454.3 | 1 | 101.062 | 434515840 | HR | MLOC_56655.1 | 3 | 57.507 | 407217240 | HR | HR-HR |
| MLOC_19686.5 | 1 | 100.850 | 432422720 | HR | MLOC_56893.7 | 3 | 57.436 | 406112400 | HR | HR-HR |
| AK250378.1 | 1 | 100.142 | 430624720 | HR | MLOC_7612.1 | 3 | 55.807 | 398771600 | HR | HR-HR |
| MLOC_72442.1 | 1 | 90.297 | 416529640 | HR | MLOC_4753.2 | 3 | 55.807 | 398771600 | HR | HR-HR |
| MLOC_44606.1 | 1 | 100.071 | 430162200 | HR | MLOC_37776.4 | 3 | 55.737 | 396782640 | HR | HR-HR |
| MLOC_36420.1 | 1 | 100.071 | 430162200 | HR | AK353559 | 3 | 55.737 | 396782640 | HR | HR-HR |
| MLOC_11569.1 | 1 | 93.123 | 422290680 | HR | MLOC_2059.2 | 3 | 56.197 | 399728880 | HR | HR-HR |
| AK356092 | 1 | 95.467 | 425396880 | HR | MLOC_19332.3 | 3 | 56.445 | 399728880 | HR | HR-HR |
| MLOC_66415.3 | 1 | 95.609 | 425396880 | HR | AK366322 | 3 | 53.258 | 375190520 | HR | HR-HR |
| AK248501.1 | 1 | 95.609 | 425396880 | HR | MLOC_64727.3 | 3 | 53.258 | 375190520 | HR | HR-HR |
| MLOC_60475.1 | 1 | 106.728 | 439756080 | HR | MLOC_62570.3 | 3 | 54.214 | 383952360 | HR | HR-HR |
| AK358866 | 1 | 106.728 | 439756080 | HR | MLOC_57388.1 | 3 | 54.214 | 383952360 | HR | HR-HR |
| MLOC_54926.1 | 1 | 106.161 | 439756080 | HR | AK250359.1 | 3 | 53.258 | 377656880 | HR | HR-HR |
| MLOC_59075.1 | 1 | 111.827 | 442557240 | HR | MLOC_12156.1 | 3 | 52.620 | 359507000 | HR | HR-HR |
| MLOC_53471.2 | 1 | 117.493 | 447276680 | HR | MLOC_12953.1 | 3 | 53.063 | 366675440 | HR | HR-HR |
| AK368508 | 1 | 116.289 | 444378800 | HR | MLOC_15173.1 | 3 | 52.940 | 364490560 | HR | HR-HR |
| MLOC_25774.1 | 1 | 117.493 | 447276680 | HR | MLOC_78236.1 | 3 | 52.940 | 364490560 | HR | HR-HR |
| MLOC_62564.2 | 1 | 117.493 | 447276680 | HR | MLOC_65417.5 | 3 | 52.940 | 364490560 | HR | HR-HR |
| MLOC_59485.1 | 1 | 117.493 | 447276680 | HR | MLOC_64487.1 | 3 | 52.940 | 364490560 | HR | HR-HR |
| MLOC_57640.2 | 1 | 118.746 | 449124280 | HR | MLOC_548.2 | 3 | 52.620 | 359137480 | HR | HR-HR |
| AK249944.1 | 1 | 119.327 | 449889360 | HR | MLOC_5835.2 | 3 | 53.258 | 374225800 | HR | HR-HR |
| MLOC_36752.2 | 1 | 119.688 | 452947200 | HR | MLOC_4609.1 | 3 | 52.443 | 356951360 | HR | HR-HR |
| AK354470 | 1 | 119.688 | 452947200 | HR | AK248363.1 | 3 | 53.258 | 367286760 | HR | HR-HR |
| AK373390 | 2 | 22.167 | 24787600 | HR | MLOC_10963.3 | 4 | 54.320 | 376982320 | HR | HR-HR |
| AK374637 | 2 | 23.159 | 25540280 | HR | MLOC_66875.1 | 4 | 52.479 | 353822840 | HR | HR-HR |
| MLOC_51199.2 | 2 | 58.782 | 444174200 | HR | MLOC_17999.1 | 6 | 56.817 | 354485000 | HR | HR-HR |
| MLOC_57706.1 | 2 | 62.465 | 473605600 | HR | MLOC_54966.1 | 6 | 55.967 | 336327680 | HR | HR-HR |
| AK363087 | 2 | 64.448 | 478643720 | HR | MLOC_67303.1 | 6 | 55.524 | 325713280 | HR | HR-HR |
| AK373368 | 2 | 66.360 | 486247400 | HR | MLOC_39632.1 | 6 | 50.850 | 87927160 | HR | HR-HR |
| AK354879 | 2 | 65.368 | 485003680 | HR | MLOC_3335.2 | 6 | 55.524 | 314590480 | HR | HR-HR |
| AK375382 | 2 | 67.918 | 491392160 | HR | MLOC_54044.1 | 6 | 55.524 | 317604920 | HR | HR-HR |
| MLOC_69815.1 | 2 | 68.555 | 493862240 | HR | MLOC_55372.2 | 6 | 56.409 | 350136320 | HR | HR-HR |
| MLOC_70754.1 | 2 | 70.822 | 499169440 | HR | MLOC_70927.2 | 6 | 56.038 | 339271440 | HR | HR-HR |
| AK248516.1 | 2 | 68.555 | 495396120 | HR | MLOC_9937.4 | 6 | 55.524 | 321202160 | HR | HR-HR |
| MLOC_17010.1 | 2 | 69.370 | 496339760 | HR | MLOC_55967.1 | 6 | 55.524 | 315132360 | HR | HR-HR |
| AK376453 | 2 | 67.918 | 492932240 | HR | MLOC_55960.2 | 6 | 55.524 | 325713280 | HR | HR-HR |
| AK370579 | 2 | 70.822 | 501617200 | HR | AK354186 | 6 | 55.524 | 320352760 | HR | HR-HR |
| AK353929 | 2 | 72.450 | 503937240 | HR | AK368657 | 6 | 56.781 | 354008840 | HR | HR-HR |
| MLOC_19204.1 | 2 | 72.592 | 503937240 | HR | AK373328 | 6 | 56.781 | 354008840 | HR | HR-HR |
| AK252600.1 | 2 | 74.150 | 509879320 | HR | MLOC_13871.1 | 6 | 56.445 | 352064520 | HR | HR-HR |
| MLOC_6366.1 | 2 | 74.292 | 512036920 | HR | MLOC_64344.3 | 6 | 57.188 | 357773480 | HR | HR-HR |
| MLOC_1940.1 | 2 | 74.292 | 512036920 | HR | MLOC_68281.1 | 6 | 57.188 | 357773480 | HR | HR-HR |
| MLOC_37227.2 | 2 | 74.292 | 512036920 | HR | AK357848 | 6 | 56.409 | 348583840 | HR | HR-HR |
| MLOC_1250.1 | 2 | 76.062 | 518715560 | HR | MLOC_5149.1 | 6 | 56.409 | 343799920 | HR | HR-HR |
| MLOC_54937.1 | 2 | 76.062 | 518715560 | HR | AK362381 | 6 | 61.686 | 418904240 | HR | HR-HR |
| MLOC_16693.3 | 2 | 76.062 | 518715560 | HR | AK373295 | 6 | 55.524 | 311966640 | HR | HR-HR |
| MLOC_61959.1 | 2 | 77.266 | 522529800 | HR | MLOC_5316.1 | 6 | 59.791 | 366789520 | HR | HR-HR |
| MLOC_77511.1 | 2 | 80.312 | 524697320 | HR | MLOC_57796.1 | 6 | 64.058 | 434276520 | HR | HR-HR |
| MLOC_57566.3 | 2 | 87.642 | 540117960 | HR | MLOC_63586.3 | 6 | 60.623 | 415634360 | HR | HR-HR |
| MLOC_44062.1 | 2 | 87.642 | 540117960 | HR | MLOC_61420.1 | 6 | 59.667 | 365736760 | HR | HR-HR |
| AK374308 | 2 | 85.977 | 535495240 | HR | MLOC_60958.1 | 6 | 60.623 | 411029000 | HR | HR-HR |
| AK371551 | 2 | 99.257 | 568310600 | HR | MLOC_70531.1 | 6 | 63.881 | 434276520 | HR | HR-HR |
| AK354763 | 2 | 107.153 | 578999400 | HR | MLOC_4843.1 | 6 | 63.456 | 434138880 | HR | HR-HR |
| MLOC_59557.1 | 2 | 107.153 | 574526720 | HR | MLOC_5565.1 | 6 | 63.456 | 434138880 | HR | HR-HR |
| MLOC_7955.1 | 2 | 107.153 | 578796040 | HR | MLOC_71541.1 | 6 | 63.881 | 434276520 | HR | HR-HR |
| AK373673 | 2 | 107.153 | 574526720 | HR | MLOC_58998.1 | 6 | 63.456 | 429255760 | HR | HR-HR |
| MLOC_45114.1 | 2 | 109.419 | 581934480 | HR | MLOC_67091.1 | 6 | 63.314 | 428314600 | HR | HR-HR |
| MLOC_54978.1 | 2 | 110.907 | 584655040 | HR | MLOC_71297.2 | 6 | 68.201 | 464273360 | HR | HR-HR |
| MLOC_54974.4 | 2 | 108.003 | 581285960 | HR | MLOC_5648.1 | 6 | 58.569 | 362022960 | HR | HR-HR |
| MLOC_62877.1 | 2 | 107.153 | 578796040 | HR | AK355424 | 6 | 60.623 | 415150760 | HR | HR-HR |
| MLOC_63401.3 | 2 | 107.153 | 578796040 | HR | MLOC_12637.2 | 6 | 60.623 | 415150760 | HR | HR-HR |
| MLOC_6839.3 | 5 | 77.083 | 440362440 | HR | MLOC_39328.2 | 7 | 69.263 | 184054440 | HR | HR-HR |
| AK353941 | 5 | 77.083 | 440362440 | HR | MLOC_37400.1 | 7 | 69.486 | 199641240 | HR | HR-HR |
| AK369670 | 5 | 80.347 | 443370680 | HR | AK364295 | 7 | 69.702 | 204524360 | HR | HR-HR |
| AK375921 | 6 | 43.768 | 32967880 | HR | AK362472 | 7 | 85.977 | 528399960 | HR | HR-HR |
| MLOC_5837.1 | 6 | 49.515 | 66692160 | HR | MLOC_54829.2 | 7 | 88.913 | 533234720 | HR | HR-HR |
| AK357603 | 6 | 50.354 | 82856800 | HR | AK366098 | 7 | 92.210 | 537045240 | HR | HR-HR |
| MLOC_1088.10 | 6 | 47.185 | 43475640 | HR | MLOC_80454.4 | 7 | 97.273 | 541751040 | HR | HR-HR |
| AK355829 | 6 | 50.850 | 84617600 | HR | AK363947 | 7 | 98.300 | 546671360 | HR | HR-HR |
| MLOC_49937.2 | 6 | 50.850 | 84084400 | HR | AK376424 | 7 | 100.024 | 549595280 | HR | HR-HR |
| MLOC_12984.1 | 6 | 74.150 | 485002440 | HR | AK366176 | 7 | 66.076 | 120241560 | HR | HR-HR |
| MLOC_15950.1 | 6 | 75.496 | 490541520 | HR | MLOC_5946.3 | 7 | 64.802 | 114654120 | HR | HR-HR |
| MLOC_75658.2 | 6 | 75.496 | 490541520 | HR | AK360925 | 7 | 64.802 | 113035920 | HR | HR-HR |
| AK376772 | 6 | 73.796 | 484391120 | HR | AK359231 | 7 | 64.802 | 114654120 | HR | HR-HR |
| MLOC_73298.1 | 6 | 72.946 | 484391120 | HR | MLOC_60854.2 | 7 | 64.164 | 111886440 | HR | HR-HR |
| MLOC_67894.2 | 6 | 73.159 | 484391120 | HR | AK360552 | 7 | 61.756 | 93226920 | HR | HR-HR |
| MLOC_58089.1 | 6 | 72.946 | 484391120 | HR | MLOC_4421.1 | 7 | 62.854 | 106987200 | HR | HR-HR |
| MLOC_76686.3 | 6 | 72.946 | 484391120 | HR | MLOC_10579.1 | 7 | 63.952 | 108338800 | HR | HR-HR |
| MLOC_12438.2 | 6 | 72.238 | 480187520 | HR | MLOC_47072.1 | 7 | 61.756 | 94112280 | HR | HR-HR |
| MLOC_7591.1 | 6 | 72.238 | 480187520 | HR | MLOC_12320.2 | 7 | 62.394 | 99911760 | HR | HR-HR |
| MLOC_80730.1 | 6 | 72.238 | 473930480 | HR | AK357813 | 7 | 62.394 | 99911760 | HR | HR-HR |
| AK372135 | 6 | 72.238 | 480187520 | HR | AK367975 | 7 | 62.783 | 103263480 | HR | HR-HR |
| MLOC_59843.1 | 6 | 72.238 | 480187520 | HR | AK376694 | 7 | 62.783 | 104746520 | HR | HR-HR |
| MLOC_51591.2 | 6 | 71.388 | 472737600 | HR | MLOC_36316.1 | 7 | 62.288 | 97527240 | HR | HR-HR |
| MLOC_70155.10 | 6 | 71.388 | 473048840 | HR | AK357227 | 7 | 62.394 | 97821120 | HR | HR-HR |
| MLOC_52134.1 | 6 | 78.116 | 494984440 | HR | MLOC_51960.1 | 7 | 62.394 | 97821120 | HR | HR-HR |
| AK371858 | 6 | 76.419 | 492633400 | HR | MLOC_63427.2 | 7 | 61.969 | 95920200 | HR | HR-HR |
| MLOC_53580.1 | 6 | 86.756 | 504258400 | HR | AK363330 | 7 | 54.816 | 77874480 | HR | HR-HR |
| AK252771.1 | 6 | 86.756 | 503835560 | HR | AK361016 | 7 | 52.762 | 73431560 | HR | HR-HR |
| AK359973 | 6 | 86.261 | 503639640 | HR | AK361067 | 7 | 56.374 | 84858160 | HR | HR-HR |
| MLOC_36834.1 | 6 | 86.261 | 500805000 | HR | AK359510 | 7 | 56.374 | 84858160 | HR | HR-HR |
| MLOC_12766.3 | 6 | 88.244 | 504848640 | HR | AK376548 | 7 | 62.394 | 99911760 | HR | HR-HR |
| MLOC_37029.1 | 6 | 88.739 | 507643600 | HR | AK248231.1 | 7 | 52.851 | 73431560 | HR | HR-HR |
| AK355803 | 6 | 95.042 | 513092160 | HR | MLOC_56157.2 | 7 | 50.708 | 71270240 | HR | HR-HR |
| MLOC_51556.2 | 6 | 94.901 | 513092160 | HR | AK372627 | 7 | 50.708 | 71270240 | HR | HR-HR |
| MLOC_57146.1 | 6 | 96.955 | 515353920 | HR | AK367641 | 7 | 50.708 | 68111960 | HR | HR-HR |
| MLOC_76165.1 | 6 | 100.425 | 516580280 | HR | MLOC_61880.1 | 7 | 48.017 | 65060320 | HR | HR-HR |
| AK358031 | 6 | 104.816 | 522565760 | HR | AK359427 | 7 | 46.388 | 60249120 | HR | HR-HR |
| AK251445.1 | 6 | 104.958 | 522565760 | HR | MLOC_47634.1 | 7 | 44.405 | 54354160 | HR | HR-HR |
| MLOC_5972.1 | 6 | 104.816 | 522565760 | HR | MLOC_52403.1 | 7 | 44.405 | 54354160 | HR | HR-HR |
| MLOC_15081.1 | 6 | 104.816 | 522565760 | HR | AK248176.1 | 7 | 41.431 | 51280200 | HR | HR-HR |
| MLOC_1297.1 | 6 | 105.312 | 522565760 | HR | MLOC_64985.8 | 7 | 41.431 | 52422240 | HR | HR-HR |
| MLOC_59134.1 | 6 | 118.980 | 533625320 | HR | MLOC_67619.2 | 7 | 20.822 | 22661000 | HR | HR-HR |
| AK252716.1 | 1 | 101.062 | 434515840 | HR | MLOC_12284.1 | 3 | 51.629 | 286996760 | Het | LR-HR |
| AK373107 | 1 | 101.062 | 434515840 | HR | MLOC_59847.1 | 3 | 51.629 | 291454560 | Het | LR-HR |
| MLOC_61455.1 | 1 | 101.062 | 434515840 | HR | MLOC_16643.2 | 3 | 51.629 | 274974960 | LR | LR-HR |
| AK362128 | 1 | 100.850 | 432422720 | HR | AK251854.1 | 3 | 51.629 | 313712560 | LR | LR-HR |
| MLOC_51867.1 | 1 | 106.161 | 439756080 | HR | AK363968 | 3 | 51.629 | 282334360 | LR | LR-HR |
| MLOC_17865.1 | 1 | 119.688 | 452947200 | HR | MLOC_2200.1 | 3 | 51.700 | 347082200 | LR | LR-HR |
| AK252170.1 | 1 | 129.391 | 458466440 | HR | MLOC_60540.2 | 3 | 51.629 | 275397800 | LR | LR-HR |
| AK369586 | 1 | 130.135 | 458611520 | HR | MLOC_68778.1 | 3 | 51.629 | 299441400 | LR | LR-HR |
| MLOC_64743.1 | 1 | 125.992 | 456083160 | HR | AK252802.1 | 3 | 51.629 | 319633560 | LR | LR-HR |
| MLOC_3613.1 | 1 | 128.045 | 457225200 | HR | AK363708 | 3 | 51.629 | 298005480 | LR | LR-HR |
| MLOC_6992.1 | 1 | 128.045 | 456498560 | HR | AK362601 | 3 | 51.629 | 274548400 | LR | LR-HR |
| AK363231 | 1 | 130.737 | 459899880 | HR | MLOC_53418.2 | 3 | 51.629 | 274548400 | LR | LR-HR |
| MLOC_1081.1 | 1 | 130.737 | 459899880 | HR | MLOC_59013.3 | 3 | 51.629 | 281334920 | LR | LR-HR |
| AK356546 | 1 | 132.365 | 462937880 | HR | MLOC_51868.2 | 3 | 51.629 | 304880040 | LR | LR-HR |
| MLOC_66503.2 | 1 | 132.365 | 462937880 | HR | AK359804 | 3 | 51.629 | 333940680 | LR | LR-HR |
| AK354960 | 1 | 132.153 | 461836760 | HR | AK250178.1 | 3 | 51.629 | 274548400 | LR | LR-HR |
| MLOC_8093.1 | 1 | 132.720 | 464051400 | HR | AK248914.1 | 3 | 51.629 | 284215440 | LR | LR-HR |
| MLOC_70079.3 | 2 | 23.159 | 25540280 | HR | MLOC_61267.1 | 4 | 52.195 | 333449640 | LR | LR-HR |
| AK372085 | 2 | 23.796 | 27224200 | HR | MLOC_45145.1 | 4 | 52.195 | 333449640 | LR | LR-HR |
| AK363931 | 2 | 29.391 | 34614600 | HR | MLOC_64008.1 | 4 | 51.877 | 322335520 | LR | LR-HR |
| MLOC_70246.1 | 2 | 29.391 | 34782000 | HR | MLOC_25508.1 | 4 | 51.416 | 276431960 | LR | LR-HR |
| MLOC_52706.1 | 2 | 29.391 | 35720680 | HR | MLOC_52114.5 | 4 | 51.877 | 312292760 | LR | LR-HR |
| MLOC_70443.1 | 2 | 30.147 | 35914120 | HR | MLOC_60937.2 | 4 | 51.416 | 300791760 | LR | LR-HR |
| MLOC_55137.2 | 2 | 30.471 | 36494440 | HR | MLOC_66623.1 | 4 | 51.416 | 261543280 | LR | LR-HR |
| MLOC_57800.1 | 2 | 30.471 | 36494440 | HR | MLOC_13484.2 | 4 | 52.443 | 351068800 | LR | LR-HR |
| AK365327 | 2 | 57.489 | 413778080 | HR | AK357081 | 6 | 52.691 | 112252240 | LR | LR-HR |
| AK369731 | 2 | 59.829 | 457940680 | HR | MLOC_12939.2 | 6 | 55.453 | 305109440 | LR | LR-HR |
| MLOC_64083.1 | 2 | 60.375 | 459102560 | HR | MLOC_77423.4 | 6 | 54.887 | 251914680 | LR | LR-HR |
| AK367505 | 2 | 62.465 | 473605600 | HR | AK364744 | 6 | 53.754 | 202346920 | LR | LR-HR |
| AK358518 | 2 | 64.873 | 480243320 | HR | MLOC_78793.2 | 6 | 53.754 | 204468560 | LR | LR-HR |
| MLOC_57516.2 | 2 | 64.826 | 479935800 | HR | AK364234 | 6 | 55.028 | 260614520 | LR | LR-HR |
| AK359655 | 2 | 62.680 | 473605600 | HR | MLOC_53804.1 | 6 | 55.028 | 258905800 | LR | LR-HR |
| AK357185 | 2 | 64.873 | 481829280 | HR | MLOC_20229.1 | 6 | 54.391 | 212759200 | LR | LR-HR |
| MLOC_69140.1 | 2 | 67.351 | 487689520 | HR | MLOC_62478.1 | 6 | 55.382 | 296284360 | LR | LR-HR |
| AK248841.1 | 2 | 72.557 | 503937240 | HR | MLOC_67067.2 | 6 | 59.915 | 377560160 | LR | LR-HR |
| MLOC_68698.1 | 2 | 77.266 | 522529800 | HR | MLOC_39554.2 | 6 | 59.915 | 371115880 | LR | LR-HR |
| MLOC_37926.2 | 2 | 80.028 | 524237280 | HR | AK373023 | 6 | 60.198 | 400487760 | LR | LR-HR |
| MLOC_15746.1 | 2 | 80.064 | 524697320 | HR | MLOC_56103.1 | 6 | 59.915 | 373538840 | LR | LR-HR |
| MLOC_64395.3 | 2 | 79.887 | 524237280 | HR | AK363858 | 6 | 59.915 | 372719200 | LR | LR-HR |
| AK355750 | 2 | 80.028 | 524237280 | HR | AK365927 | 6 | 59.915 | 374663520 | LR | LR-HR |
| MLOC_57609.1 | 2 | 79.887 | 524237280 | HR | AK365609 | 6 | 59.915 | 371893360 | LR | LR-HR |
| AK368879 | 2 | 80.595 | 527137640 | HR | MLOC_3763.3 | 6 | 60.482 | 405765200 | LR | LR-HR |
| AK375892 | 2 | 80.453 | 524884560 | HR | MLOC_60633.2 | 6 | 60.057 | 383436520 | LR | LR-HR |
| MLOC_55138.1 | 2 | 81.799 | 530119840 | HR | AK376949 | 6 | 59.915 | 380869720 | LR | LR-HR |
| MLOC_68844.2 | 2 | 87.642 | 540117960 | HR | AK363473 | 6 | 60.234 | 403474920 | LR | LR-HR |
| AK374402 | 2 | 88.527 | 543049320 | HR | MLOC_16961.1 | 6 | 60.552 | 408102600 | LR | LR-HR |
| MLOC_51546.2 | 2 | 88.527 | 543049320 | HR | MLOC_57389.2 | 6 | 59.915 | 374663520 | LR | LR-HR |
| MLOC_60218.1 | 2 | 92.776 | 558011160 | HR | MLOC_7185.1 | 6 | 59.915 | 372719200 | LR | LR-HR |
| AK358395 | 2 | 107.153 | 571768960 | HR | AK364859 | 6 | 60.411 | 404825280 | LR | LR-HR |
| AK354660 | 5 | 73.299 | 431358800 | HR | AK354534 | 7 | 70.963 | 360541160 | LR | LR-HR |
| MLOC_5724.3 | 5 | 80.347 | 445226960 | HR | AK363115 | 7 | 70.184 | 224849200 | LR | LR-HR |
| MLOC_52057.3 | 5 | 80.347 | 442089760 | HR | MLOC_53940.1 | 7 | 70.184 | 212403320 | LR | LR-HR |
| MLOC_74474.4 | 5 | 85.556 | 455656600 | HR | AK374598 | 7 | 70.184 | 214284400 | LR | LR-HR |
| AK374424 | 5 | 84.912 | 454028480 | HR | AK249904.1 | 7 | 68.951 | 181733160 | LR | LR-HR |
| MLOC_57422.1 | 5 | 85.556 | 456331160 | HR | MLOC_9968.1 | 7 | 69.051 | 181733160 | LR | LR-HR |
| MLOC_70552.1 | 5 | 85.556 | 455404880 | HR | MLOC_64692.1 | 7 | 70.149 | 211732480 | LR | LR-HR |
| AK376458 | 5 | 85.556 | 456331160 | HR | AK249482.1 | 7 | 70.680 | 283375960 | LR | LR-HR |
| MLOC_75155.1 | 5 | 85.556 | 455125880 | HR | MLOC_14017.2 | 7 | 70.432 | 238621880 | LR | LR-HR |
| MLOC_62437.1 | 5 | 83.472 | 449473960 | HR | AK248865.1 | 7 | 70.184 | 223235960 | LR | LR-HR |
| AK364206 | 5 | 83.472 | 449473960 | HR | AK251223.1 | 7 | 70.680 | 299429000 | LR | LR-HR |
| MLOC_55896.1 | 5 | 92.986 | 460149120 | HR | AK375206 | 7 | 70.680 | 263624000 | LR | LR-HR |
| MLOC_53578.1 | 5 | 95.139 | 464759440 | HR | MLOC_6413.1 | 7 | 70.680 | 261886760 | LR | LR-HR |
| MLOC_67041.1 | 5 | 95.903 | 465844440 | HR | AK376864 | 7 | 70.680 | 261886760 | LR | LR-HR |
| AK368025 | 5 | 96.597 | 468475720 | HR | MLOC_68247.1 | 7 | 70.680 | 267944160 | LR | LR-HR |
| AK355945 | 5 | 96.597 | 468475720 | HR | AK250219.1 | 7 | 70.680 | 272188680 | LR | LR-HR |
| MLOC_64838.2 | 5 | 95.903 | 465844440 | HR | MLOC_68718.1 | 7 | 70.680 | 254637720 | LR | LR-HR |
| AK358309 | 1 | 48.725 | 247854920 | LR | AK373161 | 4 | 101.983 | 524343920 | HR | LR-HR |
| AK373165 | 2 | 56.445 | 327186400 | LR | MLOC_6391.1 | 5 | 136.389 | 511348720 | HR | LR-HR |
| AK367481 | 2 | 56.516 | 357975600 | LR | AK356143 | 5 | 136.389 | 512230360 | HR | LR-HR |
| MLOC_63300.1 | 2 | 56.374 | 321081880 | LR | AK363250 | 5 | 130.903 | 506583400 | HR | LR-HR |
| MLOC_64213.4 | 2 | 56.445 | 327904360 | LR | MLOC_6875.2 | 5 | 135.069 | 510044240 | HR | LR-HR |
| MLOC_77670.1 | 2 | 56.516 | 371450680 | LR | MLOC_62889.1 | 5 | 135.347 | 510643160 | HR | LR-HR |
| MLOC_59585.1 | 2 | 56.516 | 354523440 | LR | MLOC_55658.4 | 5 | 136.250 | 510643160 | HR | LR-HR |
| MLOC_74253.1 | 2 | 56.516 | 357975600 | LR | AK373354 | 5 | 137.049 | 512720160 | HR | LR-HR |
| MLOC_14216.1 | 2 | 56.516 | 348712800 | LR | MLOC_55943.1 | 5 | 128.194 | 502704680 | HR | LR-HR |
| MLOC_11833.2 | 6 | 50.212 | 77764120 | LR | MLOC_1982.2 | 7 | 95.503 | 539297080 | HR | LR-HR |
| MLOC_20007.1 | 6 | 50.212 | 77764120 | LR | MLOC_44010.1 | 7 | 97.309 | 542097000 | HR | LR-HR |
| AK355423 | 6 | 50.212 | 77764120 | LR | MLOC_59848.1 | 7 | 97.273 | 541751040 | HR | LR-HR |
| MLOC_73208.3 | 6 | 52.904 | 121102120 | LR | AK364678 | 7 | 97.805 | 544750600 | HR | LR-HR |
| MLOC_15650.1 | 6 | 53.116 | 143978880 | LR | AK360899 | 7 | 104.816 | 557398600 | HR | LR-HR |
| MLOC_53118.1 | 6 | 52.904 | 115787480 | LR | MLOC_62231.1 | 7 | 116.076 | 567153680 | HR | LR-HR |
| MLOC_15296.1 | 6 | 52.904 | 120513120 | LR | MLOC_66206.1 | 7 | 116.076 | 565840520 | HR | LR-HR |
| AK364394 | 6 | 52.904 | 119180120 | LR | AK362876 | 7 | 118.343 | 568919440 | HR | LR-HR |
| MLOC_69397.2 | 6 | 52.904 | 126491160 | LR | MLOC_10378.2 | 7 | 118.343 | 569145120 | HR | LR-HR |
| MLOC_37849.1 | 6 | 52.904 | 121642760 | LR | MLOC_5840.2 | 7 | 119.830 | 570548800 | HR | LR-HR |
| MLOC_63430.1 | 6 | 53.329 | 161621600 | LR | AK366600 | 7 | 119.830 | 570548800 | HR | LR-HR |
| AK249870.1 | 6 | 52.904 | 120513120 | LR | AK374495 | 7 | 120.822 | 574959480 | HR | LR-HR |
| MLOC_64491.2 | 6 | 52.904 | 131374280 | LR | MLOC_63893.1 | 7 | 124.575 | 579123400 | HR | LR-HR |
| AK250507.1 | 6 | 52.904 | 122595080 | LR | MLOC_3173.4 | 7 | 124.575 | 579753320 | HR | LR-HR |
| MLOC_61311.4 | 6 | 53.754 | 199601560 | LR | MLOC_36437.1 | 7 | 126.558 | 584284280 | HR | LR-HR |
| AK372255 | 6 | 52.054 | 92667680 | LR | MLOC_71862.1 | 7 | 129.391 | 588298160 | HR | LR-HR |
| AK375989 | 6 | 53.754 | 185877240 | LR | MLOC_15779.1 | 7 | 84.566 | 521852760 | HR | LR-HR |
| AK357463 | 6 | 54.887 | 237686920 | LR | AK362610 | 7 | 84.566 | 521852760 | HR | LR-HR |
| MLOC_40001.1 | 6 | 53.754 | 185877240 | LR | MLOC_20732.1 | 7 | 84.566 | 521852760 | HR | LR-HR |
| MLOC_55923.3 | 6 | 53.754 | 188503560 | LR | MLOC_69519.3 | 7 | 85.977 | 523831800 | HR | LR-HR |
| MLOC_61987.2 | 6 | 53.612 | 165231240 | LR | MLOC_60780.1 | 7 | 85.977 | 528399960 | HR | LR-HR |
| AK362470 | 6 | 54.887 | 245064920 | LR | AK368875 | 7 | 85.977 | 528399960 | HR | LR-HR |
| AK250311.1 | 6 | 53.470 | 164499640 | LR | MLOC_43986.1 | 7 | 85.977 | 522555840 | HR | LR-HR |
| MLOC_36391.1 | 6 | 53.470 | 163895760 | LR | AK364906 | 7 | 76.753 | 483245360 | HR | LR-HR |
| MLOC_13269.2 | 6 | 52.904 | 114394960 | LR | MLOC_36325.1 | 7 | 79.780 | 511642600 | HR | LR-HR |
| AK361188 | 6 | 53.683 | 173261480 | LR | AK368539 | 7 | 76.558 | 468529040 | HR | LR-HR |
| AK353955 | 6 | 53.329 | 157523400 | LR | AK365478 | 7 | 76.558 | 477842680 | HR | LR-HR |
| MLOC_55635.2 | 6 | 53.329 | 157523400 | LR | AK363024 | 7 | 77.266 | 498124120 | HR | LR-HR |

^a^ HR = High-recombining; LR = Low-recombining.

**Table S4.** Gene ontology terms enriched in barley ohnologs

| GO Term | Number of genes | | p value | FDR^a^ | Percentage^b^ | Genes |
| --- | --- | --- | --- | --- | --- | --- |
|  | Ohnolog | Total |  |  |  |  |
| protein S/T phosphatase complex | 8 | 81 | <0.001 | 0.026 | 9.9 | MLOC_3173, MLOC_54926, AK250359, AK363024, AK250507, AK362128, AK251854, MLOC_55635 |
| clathrin coat | 8 | 27 | <0.0000001 | 0.000 | 29.6 | MLOC_64727, MLOC_47634, MLOC_64985, AK251445, AK248501, MLOC_37776, MLOC_1297, MLOC_44606 |
| intracellular signaling cascade | 34 | 657 | <0.0001 | 0.005 | 5.2 | MLOC_54937, AK356379, MLOC_64743, AK365478, MLOC_55923, MLOC_60840, MLOC_44010, AK362892, MLOC_57706, MLOC_77423, MLOC_19561, MLOC_69519, AK252802, MLOC_63743, AK363838, AK355945, MLOC_57640, AK368252, AK362381, AK251854, AK353955, MLOC_64008, MLOC_548, MLOC_54966, MLOC_64083, AK250219, MLOC_57516, AK364234, MLOC_4609, MLOC_54274, MLOC_20007, MLOC_4800, MLOC_36752, AK363931 |
| stomatal movement | 9 | 82 | <0.001 | 0.024 | 11.0 | AK363838, AK368020, MLOC_77423, MLOC_5972, MLOC_4800, AK363457, AK251854, MLOC_52403, MLOC_64083 |
| phosphoinositide phosphorylation | 7 | 53 | <0.0001 | 0.033 | 13.2 | MLOC_56148, MLOC_59843, AK368951, MLOC_71567, MLOC_51655, AK372135, AK367975 |
| Meristem identity | 6 | 42 | <0.001 | 0.044 | 14.3 | AK249904, AK369282, AK374424, MLOC_45145, AK372085, AK368348 |

^a^ FDR; False discovery rate

^b^ [Ohnologs with GO term x 100]/ [genes with GO term]

**Table S5. Analysis of ohnolog gene expression bias by ohnolog region**

| Barley ohnolog regions  (query chromosome *vs* hit chromosome) ^a^ | Number of genes with higher expression level | | Total | Bias (Hit/Total) % |
| --- | --- | --- | --- | --- |
|  | Query | Hit |  |  |
| **3 *vs* 1** | 36 | 43 | 79 | 54 |
| **1 *vs* 4** | 3 | 4 | 7 | 44 |
| **2 *vs* 4** | 5 | 1 | 6 |  |
| **2 *vs* 5** | 2 | 3 | 5 |  |
| **2 *vs* 6** | 21 | 13 | 34 | 46 |
| **7 *vs* 6** | 18 | 20 | 38 |  |
| **5 *vs* 7** | 6 | 5 | 11 | 45 |
| **Total** | 91 | 89 | 180 |  |

^a^ Ohnologous chromosome regions are named according to their parent chromosomes and colour-coded according to Figure S9B. For more information see Figure 4, Figures S9 and S10 and Tables S1 and S3.

**Table S6. Distributions of ohnologs and ohnolog pairs for Brachypodium, rice and maize by genome compartment**

| Species | Type | Genome compartment | | Number | |
| --- | --- | --- | --- | --- | --- |
|  |  |  |  | Obs^a^ | Exp^a^ |
|  |  |  |  |  |  |
| Brachypodium | Ohnolog | HR | | 2392 | 2336 |
|  |  | LR | | 44 | 100 |
|  | Ohnolog pair | HR-HR | | 1174 | 1120 |
|  |  | LR-HR | LR | 44 | 96 |
|  |  |  | HR |  |  |
|  |  | LR-LR | | 0 | 2 |
| Rice | Ohnolog | HR | | 4954 | 4701 |
|  |  | LR | | 52 | 306 |
|  | Ohnolog pair | HR-HR | | 2451 | 2206 |
|  |  | LR-HR | LR | 52 | 287 |
|  |  |  | HR |  |  |
|  |  | LR-LR | | 0 | 9 |

**^a^** Obs, Observed; Exp, Expected (see text)

**Table S7. Brachypodium ohnolog MCScanX data**

Available as separate excel spreadsheet

**Table S8. Rice ohnolog MCScanX data**

Available as separate excel spreadsheet

**Table S9. RNA-seq data, with gene assignations and map positions**

Available as separate excel spreadsheet

**Table S10. Shared synteny blocks between Brachypodium and barley genomes**

| **Synteny Block** | **Barley chromosome** | **BARLEY cM start** | **BARLEY cM end** | **Brachypodium chromosome** | **Brachypodium gene order (Ascending/ Descending)** | **Brachypodium gene at start of synteny block** | **Barley paralog at start of synteny block** | **Brachypodium gene at end of synteny block** | **Barley paralog at end of synteny block** |
| --- | --- | --- | --- | --- | --- | --- | --- | --- | --- |
| 1.01 | 1 | 0.000 | 17.458 | 2 | D | 2.40077 | AK354945 | 2.3873 | MLOC_4383.1 |
| 1.02 | 1 | 17.847 | 47.521 | 2 | A | 2.37166 | MLOC_70910.1 | 2.39137 | MLOC_48495.2 |
| 1.03 | 1 | 47.238 | 53.683 | 3 | A | 3.2668 | AK364703 | 3.3434 | AK249402.1 |
| 1.04 | 1 | 53.754 | 65.545 | 2 | D | 2.2788 | AK370392 | 2.2255 | MLOC_45271.1 |
| 1.05 | 1 | 65.658 | 88.881 | 2 | A | 2.2057 | AK249744.1 | 2.2441 | AK372941 |
| 1.06 | 1 | 90.297 | 132.773 | 2 | D | 2.19326 | AK251409.1 | 2.14147 | MLOC_37532.2 |
| 2.01 | 2 | 0.000 | 6.303 | 5 | D | 5.0189 | AK370295 | 5.008 | MLOC_71231.2 |
| 2.02 | 2 | 6.707 | 18.909 | 5 | A | 5.0195 | AK356013 | 5.05225 | MLOC_58149.1 |
| 2.03 | 2 | 22.167 | 56.976 | 1 | A | 1.16355 | MLOC_20213.2 | 1.5964 | MLOC_55672.1 |
| 2.04 | 2 | 55.524 | 82.153 | 5 | A | 5.0462 | MLOC_81820.1 | 5.0787 | AK368461 |
| 2.05 | 2 | 82.330 | 92.776 | 5 | D | 5.1909 | AK375429 | 5.1754 | MLOC_57710.1 |
| 2.06 | 2 | 93.555 | 111.261 | 5 | D | 5.2165 | AK248312.1 | 5.2005 | MLOC_20358.1 |
| 2.07 | 2 | 111.809 | 114.235 | 5 | A | 5.2243 | MLOC_13115.2 | 5.2278 | MLOC_6059.2 |
| 2.08 | 2 | 114.377 | 134.844 | 5 | D | 5.2485 | MLOC_63185.1 | 5.2293 | MLOC_19226.1 |
| 2.09 | 2 | 135.623 | 141.941 | 5 | D | 5.2642 | MLOC_6620.3 | 5.2586 | MLOC_36886.1 |
| 2.10 | 2 | 142.341 | 149.363 | 5 | A | 5.2654 | AK362438 | 5.27687 | MLOC_76421.1 |
| 3.01 | 3 | 0.000 | 81.799 | 2 | A | 2.0059 | MLOC_34369.1 | 2.5574 | AK367531 |
| 3.02 | 3 | 83.074 | 102.975 | 2 | D | 2.5674 | MLOC_15557.1 | 2.5378 | AK362439 |
| 3.03 | 3 | 103.116 | 109.844 | 2 | D | 2.5805 | MLOC_73299.1 | 2.5679 | MLOC_64490.3 |
| 3.04 | 3 | 109.844 | 155.028 | 2 | A | 2.5811 | MLOC_4215.8 | 2.6279 | MLOC_52632.3 |
| 4.01 | 4 | 0.814 | 1.133 | 1 | D | 1.1345 | AK251104.1 | 1.00237 | MLOC_65537.6 |
| 4.02 | 4 | 1.133 | 3.470 | 4 | D | 4.4485 | MLOC_57082.1 | 4.38387 | MLOC_20887.1 |
| 4.03 | 4 | 3.470 | 44.901 | 1 | D | 1.1333 | MLOC_72361.2 | 1.093 | MLOC_59370.2 |
| 4.04 | 4 | 44.935 | 49.858 | 1 | D | 1.14307 | AK372791 | 1.1432 | AK371793 |
| 4.05 | 4 | 49.858 | 51.416 | 4 | A | 4.1571 | MLOC_56190.2 | 4.2154 | MLOC_73350.2 |
| 4.06 | 4 | 51.416 | 69.334 | 1 | A | 1.1535 | AK354903 | 1.7153 | MLOC_9879.3 |
| 4.07 | 4 | 71.990 | 88.075 | 1 | D | 1.7462 | MLOC_6726.1 | 1.7188 | MLOC_72140.1 |
| 4.08 | 4 | 91.183 | 115.227 | 1 | A | 1.7186 | AK360340 | 1.7821 | AK251005.1 |
| 5.01 | 5 | 0.000 | 43.611 | 4 | A | 4.0023 | MLOC_73572.1 | 4.0814 | MLOC_74892.1 |
| 5.02 | 5 | 44.236 | 47.014 | 4 | A | 4.3812 | MLOC_72869.1 | 4.45397 | MLOC_59716.2 |
| 5.03 | 5 | 46.875 | 47.222 | 4 | A | 4.0816 | MLOC_3165.1 | 4.08917 | MLOC_51364.3 |
| 5.04 | 5 | 47.153 | 52.292 | 4 | A | 4.2697 | MLOC_45032.1 | 4.2998 | MLOC_4614.1 |
| 5.05 | 5 | 62.500 | 51.597 | 4 | D | 4.3 | MLOC_20078.1 | 4.3155 | MLOC_54496.1 |
| 5.06 | 5 | 62.500 | 114.861 | 4 | A | 4.3156 | AK357020 | 4.3809 | MLOC_76213.1 |
| 5.07 | 5 | 117.986 | 122.431 | 1 | A | 1.1351 | AK375101 | 1.149 | MLOC_27713.1 |
| 5.08 | 5 | 122.483 | 169.375 | 1 | D | 1.08907 | MLOC_52486.1 | 1.0045 | AK368335 |
| 6.01 | 6 | 0.000 | 69.263 | 3 | A | 3.0033 | AK367861 | 3.5683 | MLOC_38384.1 |
| 6.02 | 6 | 70.007 | 94.228 | 3 | D | 3.6 | MLOC_69012.1 | 3.56087 | MLOC_45258.1 |
| 6.03 | 6 | 94.618 | 126.629 | 3 | A | 3.5363 | MLOC_18670.1 | 3.6085 | MLOC_62397.4 |
| 7.01 | 7 | 0.212 | 68.059 | 1 | D | 1.5246 | MLOC_75066.1 | 1.41907 | MLOC_38257.2 |
| 7.02 | 7 | 68.059 | 74.292 | 3 | D | 3.1505 | MLOC_12375.7 | 3.12902 | MLOC_37025.2 |
| 7.03 | 7 | 74.292 | 140.935 | 1 | D | 1.4185 | MLOC_64066.1 | 1.2913 | AK365764 |
