# Supplementary material for: The low-recombining pericentromeric region of barley restricts gene diversity and evolution but not gene expression
Source: Plant J. 2014 Aug 5;79(6):981–92. doi: 10.1111/tpj.12600 (PMC4309411; doi:10.1111/tpj.12600)
Supplement: Appendix S1 — MCScanX analysis. [file tpj0079-0981-sd6.docx]

**Appendix S1. MCScanX analysis**

MCScanX requires a unique map position for each gene, so for barley pseudo-physical genomic locations were assigned relative to the pseudo-physical location (IBSC 2012) of the synteny block containing it as follows: The lowest barley contig physical location in each block was assigned to the first gene in the synteny block, the highest barley contig physical location was assigned to the last gene in the block and the other genes in the block were assigned evenly spaced pseudo-physical locations between. For Brachypodium and rice the physical genome data (Bdistachyon_192 and Osativa_120, downloaded from [www.phytozome.net](http://www.phytozome.net)/) were used.

MCScan X was run with default parameters in all cases, apart from match size (the minimum number of neighbouring genes required to call a collinear block) that was set to three. The MCScan X output gene lists, together with the handpicked output gene sets, were inputted into MACSE (Ranwez et al. 2012) to align the putative coding sequences of the ohnolog pairs. Default parameters were used for MACSE, apart from frame shift penalty which was set to -10.

**References**

**Ranwez V., Harispe S., Delsuc F., Douzery E.J.P.** (2011). MACSE: Multiple alignment of coding sequences accounting for frameshifts and stop codons. *PLoS ONE* 69, e22594. DOI:10.1371/journal.pone.0022594.

**Okonechnikov K., Golosova O., Fursov M.** (2012). Unipro UGENE: a unified bioinformatics toolkit. *Bioinformatics* 28, 1166-1167.
